# Machine Learning for Screening Bioactive Compounds in Traditional Chinese Medicine: Computational Prediction, Multi-Omics Integration, and Clinical Translation Potential

**DOI:** 10.3390/ph19101567

**Published:** 2026-10-02

**Authors:** Zi-Meng Qi, Jing-Zhen Wu, Pan Li, Tian-Yang Xu, Peng Yu

**Affiliations:** 1 School of Pharmaceutical Sciences, Changchun University of Chinese Medicine, Changchun 130117, China; 25204897138@ccucm.edu.cn (Z.-M.Q.); 25204897140@ccucm.edu.cn (J.-Z.W.); 25104800147@ccucm.edu.cn (P.L.); 2 Innovation Practice Center, Changchun University of Chinese Medicine, Changchun 130117, China

**Keywords:** machine learning, traditional Chinese medicine, bioactive compound screening, molecular representation learning, network pharmacology, multi-omics integration, generative molecular design, drug repurposing, out-of-distribution generalization

## Abstract

Identifying bioactive compounds in traditional Chinese medicine (TCM) requires computational prioritization to be connected with chemical and pharmacological evidence. This structured narrative and critical review examines machine learning applications in compound prediction, formula-compatibility analysis, multi-omics integration, and drug development and repurposing. Molecular fingerprints, graph and sequence representations, knowledge graphs, and generative models are discussed in relation to their intended endpoints and validation conditions. Studies are classified along two independent dimensions: application scope, distinguishing direct TCM applications from natural-product studies and general drug-discovery methods, and validation level, ranging from computational prediction to chemical, experimental, and clinical evidence. Three case analyses illustrate a TCM-specific graph-learning platform with chemical confirmation, a downstream animal and metabolomics study without machine learning screening, and a proposed causal framework assembled from separate studies. Across these applications, machine learning supports candidate ranking and hypothesis generation, but predicted targets, attention weights, and multi-omics associations do not independently establish target engagement, formula synergy, or therapeutic efficacy. Major limitations include chemical-domain shift, inconsistent database provenance, data leakage, sparse negative results, and incomplete experimental follow-up. Reliable advancement requires scaffold-aware or external evaluation, explicit applicability domains and uncertainty, traceable data processing, and constituent-resolved intervention studies. A minimum reporting checklist consolidates these requirements, while route-specific assessment distinguishes synthetic accessibility from biosynthetic feasibility. Together, these considerations provide a basis for evaluating screening claims and designing reproducible workflows toward experimentally supported TCM compound discovery.

## 1. Introduction

Identifying bioactive compounds from traditional Chinese medicine (TCM) connects empirical clinical practice with molecular pharmacology. The practical objectives are to prioritize pharmacologically relevant constituents from single herbs or complex formulae, test their activities, and identify candidates for further drug discovery. Bioactivity-guided isolation remains important but is labor-intensive and difficult to scale across multicomponent systems [1,2]. Between 1981 and 2019, natural products contributed substantially to approved small-molecule drugs [3]. This precedent motivates computational prioritization, but it does not by itself validate predicted targets, constituent interactions, or therapeutic effects in TCM formulae.

Artificial intelligence can support TCM bioactive-compound research when its role is matched to the available evidence. Machine learning models can prioritize candidates within large chemical spaces [4,5,6,7,8], while graph and multi-omics methods can organize associations across constituents, targets, pathways, and phenotypes [9]. These outputs remain predictions or associations until they are supported by chemical confirmation and experimental intervention. This distinction is essential for multicomponent herbal systems, in which observed outcomes may reflect several interacting constituents and context-dependent exposures.

The transfer of machine learning to TCM also presents domain-specific risks. Natural products differ from many synthetic training libraries in scaffold diversity, stereochemistry, and metabolic fate [10]. Sparse standardized bioactivity measurements further limit reliable out-of-distribution prediction. In formula research, co-occurrence and network proximity identify associations, but they do not establish pharmacological interaction or causal regulation without appropriate experiments. These boundaries motivate the scope and evidence grading used throughout this review.

This structured narrative and critical review organizes the selected literature around TCM bioactive-compound screening, formula analysis, multi-omics evidence, and drug-development support. We use three application-scope tiers throughout. Tier I denotes methods or studies directly applied to TCM data, formulae, constituents, or interventions. Tier II denotes methods validated in herbal-medicine or natural-product settings but have not yet demonstrated TCM-specific screening. Tier III denotes general drug-discovery methods whose relevance to TCM remains prospective. Scope tier is independent of evidence level. The latter is graded from computational prediction through chemical confirmation, biochemical testing, cellular validation, animal studies, human observation, and clinical evidence. Supplementary Table S1 applies both classifications to the case studies and representative examples. Bibliographic traceability information for the reviewed sources is provided in Supplementary Table S3.

The reviewed evidence spans computational prioritization, downstream experimental validation, and prospective causal integration. The three cases were selected to expose different positions in this evidence chain. Case Study I is a Tier I machine learning framework with chemical confirmation but predominantly predicted targets. Case Study II is a Tier I downstream animal and metabolomics study that contains no machine learning screening step. It is retained as a boundary case for experimental validation. Case Study III is a proposed framework assembled from separate studies and is not presented as a completed causal demonstration. These examples represent different validation levels and are therefore not methodologically equivalent.

**Figure 1:**
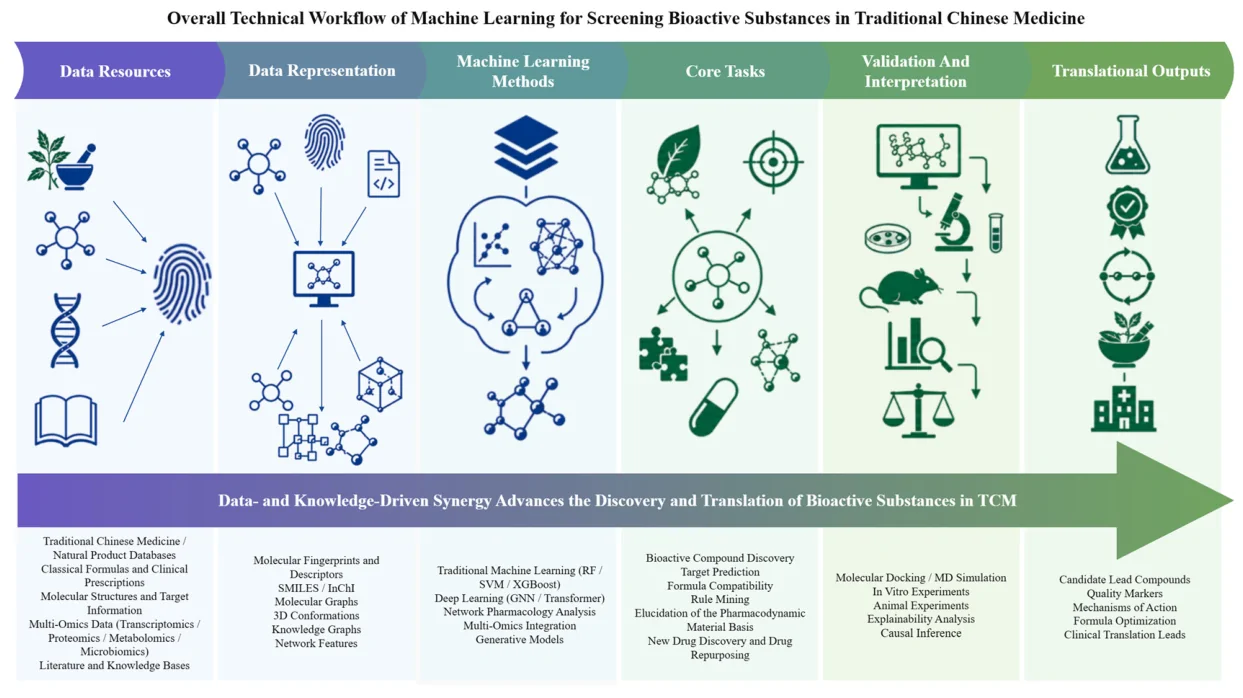
Bounded workflow for machine learning-supported bioactive-compound research in TCM. Data resources are converted into molecular, graph, or multi-omics representations and used for candidate prioritization or association analysis. Computational outputs require staged validation. The evidentiary sequence proceeds from prediction to chemical confirmation, biochemical testing, cellular validation, animal studies, human observation, and clinical evidence. Not every study completes every stage, and later stages must not be inferred from earlier computational performance.

The review therefore focuses on how machine learning can support TCM bioactive-compound discovery and how such predictions should be evaluated. Mathematical principles are retained only where they clarify model assumptions, transferability, or evidentiary limits. The principal synthesis concerns validation design, database provenance, and the transition from computational ranking to experimentally supported conclusions. Figure 1 summarizes this bounded workflow, while Supplementary Table S2 records the dataset and validation context for selected quantitative performance statements. The source context for selected numerical claims is provided in Supplementary Table S10. A source-grounded comparison with recent TCM-AI reviews [11,12,13,14,15,16,17,18] is provided in Supplementary Table S11. The distinguishing contribution of the present review is its explicit separation of application scope from validation level, together with operational reporting for database provenance, leakage and split integrity, claim boundaries, and route-specific synthetic and biosynthetic feasibility.

Database choice is a major source of analytical heterogeneity in TCM research. A nine-database assessment found only 78 shared herbal entries, while the highest average Jaccard index among seven comparable resources was 0.16 [19]. These differences can change the chemical universe available to network or machine learning analyses. Reporting of database versions, identifiers, mapping, preprocessing, deduplication, and unresolved records was inconsistent across studies, limiting reproducibility. When feasible, investigators should repeat key analyses across more than one database and report whether the principal findings remain stable, as summarized in the section entitled “Minimum Reporting Checklist”. Key TCM concepts used throughout this review are defined in Box 1.

Supplementary Table S4 separates publication and release snapshots, entity definitions, and counting rules for representative databases. Totals should be compared only after aligning the counted entity and snapshot.

Box 1 **Operational glossary of core TCM concepts for non-specialist readers.**

**Sovereign, minister, assistant, and courier (Jun-Chen-Zuo-Shi).** The hierarchical role system of a TCM formula. The sovereign herb addresses the principal pathology, minister herbs reinforce it, assistant herbs modulate or restrain, and courier herbs harmonize the formula or direct it toward specific tissues.

**Four qi.** The thermal property classification of herbs (cold, hot, warm, or cool), approxi-mately corresponding to observed effects on metabolic and inflammatory tone.

**Five flavors.** The taste-based property classification (pungent, sweet, sour, bitter, or salty), used as a proxy for pharmacological tendencies.

**Meridian tropism (guijing).** The traditional designation of the organ systems toward which a herb’s effects are directed; in computational models, it is treated as a tissue- or pathway-targeting prior.

**Syndrome (zheng).** A pattern-based diagnostic unit that groups signs and symptoms, roughly analogous to a clinical endophenotype.

**Clearing heat and purging fire.** A functional category corresponding approximately to anti-inflammatory and antipyretic actions.

**Intermingled stasis and heat.** A post-traumatic syndrome combining microcirculatory stasis with inflammatory activation.

**Eighteen Incompatible Pairs (Shiba Fan).** A classical list of herb pairs considered contra-indicated because of toxicity or antagonism.

## 2. Computational Discovery of Bioactive Compounds

### 2.1. Molecular Representations for Candidate Prioritization

#### 2.1.1. Molecular Fingerprints and Descriptor-Based QSAR

Descriptor-based quantitative structure–activity relationship models relate measured activity to specified structural or physicochemical features. Their coefficients and predictions depend on the endpoint, training domain, and functional form; a fitted relationship should not be interpreted as a universal law of natural-product activity [20,21,22,23,24].

Extended-connectivity fingerprints (ECFP, also called circular or Morgan-type fingerprints) iteratively encode atom-centered neighborhoods and can be folded into fixed-length vectors. The radius, vector length, count/bit representation, and collision handling are implementation choices rather than universal constants; variation in these descriptors across studies limits direct comparison. These settings should therefore be reported for each study. When paired with methods such as random forests or support vector machines, these descriptors provide useful baselines, but performance depends on endpoint, chemical-space coverage, split design, and tuning [20,21,22,23,24,25]. Natural-product applications are especially vulnerable to scaffold bias, out-of-distribution structures, and activity cliffs [10,24].

#### 2.1.2. Molecular Graphs and Three-Dimensional Representations

Molecular graph models represent atoms and bonds as nodes and edges. Message passing updates an atom representation using information aggregated from its neighbors. A generic update can be written as:

$$m_{v}^{\left(t+1\right)}=\sum_{u\in N\left(v\right)}M_{t}\left(h_{v}^{\left(t\right)},h_{u}^{\left(t\right)},e_{vu}\right)\;h_{v}^{\left(t+1\right)}=U_{t}\left(h_{v}^{\left(t\right)},m_{v}^{\left(t+1\right)}\right)$$

where $M_{t}$ is a message function, and $U_{t}$ is an update function. The message-passing framework learns molecular representations through neighborhood aggregation and update functions [26]. These representations do not themselves establish physical interactions or pharmacological mechanisms [20,23].

Uni-Mol is a Tier III general drug-discovery model rather than a TCM-validated system. For binding-pose prediction, the study was fine-tuned on the PDBbind General Set v2020 after excluding complexes similar to the CASF-2016 benchmark. CASF-2016, comprising 285 protein–ligand complexes, served as the held-out benchmark. Uni-Mol reported success rates of 70.53% and 88.07% below 1.0 and 2.0 Å root-mean-square deviation, compared with 44.21% and 64.56% for AutoDock Vina version 1.2.0 with exhaustiveness set to 8 [27]. The cited supplementary benchmark Table does not provide confidence intervals for these percentages. These values describe one dataset and split and should not be generalized to structurally distinct natural products. Q-GEM likewise reported gains on study-specific MoleculeNet tasks [28]. The dataset and validation context are summarized in Supplementary Table S2.

Self-supervised pretraining has expanded the scale of general molecular representation learning. GROVER used 10 million unlabeled molecules [29] and evaluated 11 MoleculeNet tasks with scaffold-balanced splits, while MolFormer-XL was pretrained on 1.1 billion SMILES strings [30]. CRA evaluated few-shot performance on MoleculeNet and FS-Mol and reported a 2.60 percentage-point AUC improvement under its benchmark protocol [31]. These Tier III results were obtained with different endpoints, splits, metrics, and comparators. They are therefore presented as within-study findings rather than a cross-model ranking. Transfer to TCM or structurally unusual natural products remains unverified in these benchmarks [10,32,33]. Supplementary Table S2 records the available evaluation context.

#### 2.1.3. Molecular Sequence and Chemical Language Models

Molecules can also be represented as SMILES or InChI sequences. Transformer models such as ChemBERTa and MolBERT learn contextual representations from these strings, but most reported benchmarks remain Tier III evidence from general molecular datasets. Image and spectroscopy models for herbal authentication or quality grading [34,35,36,37,38,39,40,41,42,43,44,45,46] are classified separately as Tier I or Tier II contextual applications. They do not constitute evidence of bioactive-compound screening unless the analysis links the material to a verified constituent or activity endpoint.

Chemical language models accommodate variable-length inputs and can reuse knowledge from large unlabeled corpora. However, multiple SMILES strings can represent one molecule, and sequence encodings do not fully resolve three-dimensional conformation or chemical feasibility. Non-destructive spectroscopy and imaging can support authentication and quality assessment [47,48,49,50,51,52,53], but these tasks remain outside the core evidence chain for activity prediction unless they are connected to chemically and pharmacologically verified endpoints.

Table 1 distinguishes the information encoded by representative molecular representations and the conditions that limit their use. Two- and three-dimensional representations can incorporate information unavailable from a single sequence, but additional structural detail or larger pretraining corpora do not guarantee better transfer. Models intended for TCM should be evaluated on relevant natural-product scaffolds and endpoints under matched validation protocols [10,24,32,33].

### 2.2. Model Selection and Screening Reliability

No fixed sample-size threshold or universally dominant model family determines performance in bioactive-compound screening. Model choice is jointly shaped by the prediction endpoint, effective sample size, chemical and biological diversity, molecular representation, split strategy, hyperparameter search, and the required form of interpretation [55,56]. Graph neural networks can be advantageous when graph structure supplies a useful inductive bias and the data support its complexity; pretrained sequence models may help when transfer is well matched to the target chemical space; and descriptor-based tree ensembles can remain competitive in smaller or structured datasets. These are general tendencies, not ranking rules. Candidate models should be compared under the same endpoint, data partition, preprocessing, and tuning budget, with scaffold-aware or external evaluation used when extrapolation is the intended claim [10,23,24,32,33,57].

In TCM applications, predictive reliability depends on external transfer to relevant natural-product scaffolds, endpoint quality, activity cliffs, and the applicability domain. A representation that performs well on an original benchmark still requires chemical confirmation and experimental follow-up in the intended screening setting.

### 2.3. From Target Prediction to Pathway Hypotheses

In network-pharmacology workflows, machine learning supports target prediction, pathway enrichment, and network-topology analysis [58,59]. Most outputs remain Tier I computational predictions when applied to TCM data, because target occupancy, intracellular exposure, and pathway crosstalk are rarely measured [10,60]. Consensus prediction may improve internal robustness [61], but it does not substitute for external or experimental validation. Claims in this section are therefore restricted to predicted associations unless a cited study reports direct testing.

Prospective virtual-screening hit rates should be interpreted in relation to the target, library composition, assay threshold, and confirmation procedure rather than equated with retrospective benchmark accuracy. Natural-product screening adds further uncertainty because standardized activity measurements and negative results remain sparse [10]. Supplementary Table S2 separates computational benchmarks, held-out tests, and prospective or experimental confirmation.

### 2.4. Virtual Screening: Throughput and Feasibility

SSnet combines protein-backbone curvature and torsion features with Morgan fingerprints for protein–ligand interaction prediction [62]. The study trained and tested models on study-specific experimental BindingDB data and also evaluated DUD-E and organism-specific datasets. Its throughput claim of one million interaction scores in approximately 18 min was measured on an NVIDIA P100 GPU. This hardware benchmark is distinct from predictive validation and should not be read as evidence of natural-product or TCM generalization. Evaluation context is listed in Supplementary Table S2.

**Figure 2:**
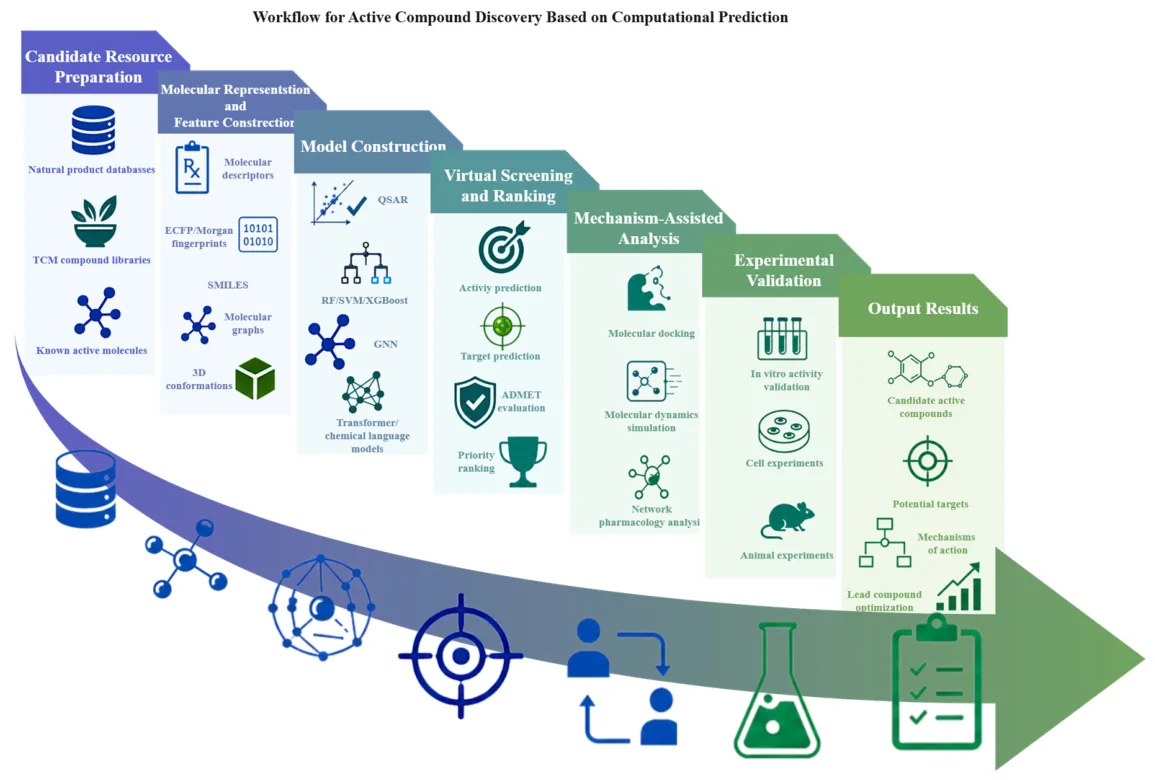
Evidence-aware workflow for computational prioritization of active compounds. Candidate resources are represented with molecular descriptors, fingerprints, sequences, graphs, or three-dimensional conformations. Models generate ranked predictions that require applicability-domain assessment, artifact filtering, chemical confirmation, and experimental testing. Molecular docking and pathway analysis provide supporting hypotheses, not independent proof of target engagement or therapeutic effect.

Candidate prioritization should also consider how a molecule can be obtained. Synthetic accessibility and biosynthetic feasibility address different acquisition constraints and do not establish biological activity; the route-specific distinction is developed in Section 5.2 [63,64,65]. Figure 2 summarizes the workflow for computational prioritization and subsequent experimental validation.

## 3. Intelligent Mining of Formula Compatibility Principles

### 3.1. Network Pharmacology and Knowledge Graph Foundations

#### 3.1.1. Network Pharmacology and Computational Implementation

Network pharmacology represents constituent, target, and pathway relationships as associations to be tested [66,67]. The network target-based identification of multicomponent synergy (NIMS) approach uses network topology and agent-related information to prioritize combinations, with selected predictions evaluated in their original experimental setting [68]. Other formula and constituent studies identify candidate targets or pathways [69,70,71]. Evidence from a tested combination cannot be transferred automatically to untested herb pairs.

#### 3.1.2. Knowledge Graph Construction and Embedding

Knowledge graphs (KGs) provide a structured representational framework for the complex knowledge system of TCM [72]. OpenTCM extracts 3.73 million characters from 68 classical gynecological texts to construct a multi-relational knowledge graph comprising over 48,000 entities and 152,000 relational triples [73]. TCM-MKG, designed specifically for formula compatibility analysis, integrates more than 30 authoritative data sources and aligns with international standards, including ICD-11, UMLS, and MeSH [74]. In addition, next-generation databases such as TCMM and ETCM organize herb–ingredient–target–disease associations through multi-relational graph structures [75,76]. A central challenge in KG construction lies in terminology standardization. The TCM conceptual system contains extensive ambiguous and overlapping content, and the prevalence of synonymous and polysemous terms substantially increases the difficulty of entity alignment.

The HTINet2 framework combines knowledge graph embedding with a residual-like graph convolutional network for herb–target ranking [77]. The study divided 38,002 known herb–target relationships into training, validation, and test sets in an 8:1:1 ratio. On the held-out test set, HTINet2 reported HR@10 of 0.4458 and NDCG@10 of 0.3746, corresponding to relative gains of approximately 122.8% and 35.7% over the best baseline. These figures are specific to the study’s random relation split and do not establish performance on unseen herbs, novel scaffolds, or external TCM databases. Details are summarized in Supplementary Table S2.

Table 2 separates databases, analysis software, and predictive models. Their outputs answer different questions: visualization displays an input network; enrichment tests annotation overrepresentation; and graph learning predicts relationships or categories. None of these outputs alone establishes a causal mechanism. Domain-specific resources can improve terminology alignment, but transferability and independent evidence support must still be evaluated.

### 3.2. Association Rules and Frequent Itemsets

Association-rule mining summarizes recurring combinations in prescription collections. The cited depression-formula analysis identified 21 rules in a 270-herb dataset [84]; related studies examine recurrent combinations in other prescription collections [85]. Support and confidence describe co-occurrence, which can reflect shared indications or prescribing convention rather than pharmacological interaction. Apriori and FP-Growth have different computational strategies, but any speed comparison requires matched datasets, thresholds, implementations, and hardware. Mined patterns therefore generate hypotheses for experimental testing.

### 3.3. Network Topology and Candidate Combinations

Network analysis extends frequent-item mining by representing herb co-occurrence and candidate biological relations as graphs. Topological and agent scores can summarize network position and prioritize herb combinations, but static network proximity does not establish a beneficial interaction, feedback process, temporal response, or dose dependence. Analyses of large prescription collections can reveal recurrent structures and clinical-use associations; they require constituent-level and intervention-based testing before pharmacological conclusions are drawn.

### 3.4. Graph Learning and Attention-Based Formula Analysis

GraphAI-for-TCM integrates a multidimensional knowledge graph built around 6080 formulae with neighbor diffusion and graph-attention modeling [74]. The primary study reports 343,373 recorded TCM compound–target associations before diffusion and 6,526,940 retained associations after diffusion at a model-predicted pKi > 5.0 threshold, equivalent to predicted K_i_ < 10 μM. The 6,183,567 associations added computationally therefore comprise 94.74% of the final set and are model-generated links, not direct measurements. This threshold is a model-derived filter rather than biochemical confirmation. Virtual nodes and attention weights summarize learned Jun-Chen-Zuo-Shi patterns, while UPLC-QToF-MS confirms detected chemical constituents but not the predicted compound–target edges. Target, pathway, attention, and herb-pair outputs remain hypotheses rather than evidence of pharmacological interaction, treatment efficacy, or clinical utility (Supplementary Table S5).

Formula graphs may encode herb co-occurrence, categorical medicinal properties, or curated relations at different levels of biological detail [86,87,88]. Such encodings can summarize empirical patterns without resolving constituent structures, exposures, or target engagement. The correspondence between traditional property categories and molecular mechanisms therefore remains a question for explicit testing rather than an assumption built into the interpretation.

### 3.5. Formula Interaction Prediction and Safety Constraints

Machine learning can rank putative interactions, but interpretation depends on validation. NIMS illustrates network-based combination prioritization with experimental follow-up in its original setting [68]. Graph models can quantify formula patterns and model-derived Jun-Chen-Zuo-Shi roles [74]. Prescription-oriented systems [89,90,91,92,93,94] require additional evaluation of contraindications, dose limits, and toxicity before their recommendations can inform practice.

**Figure 3:**
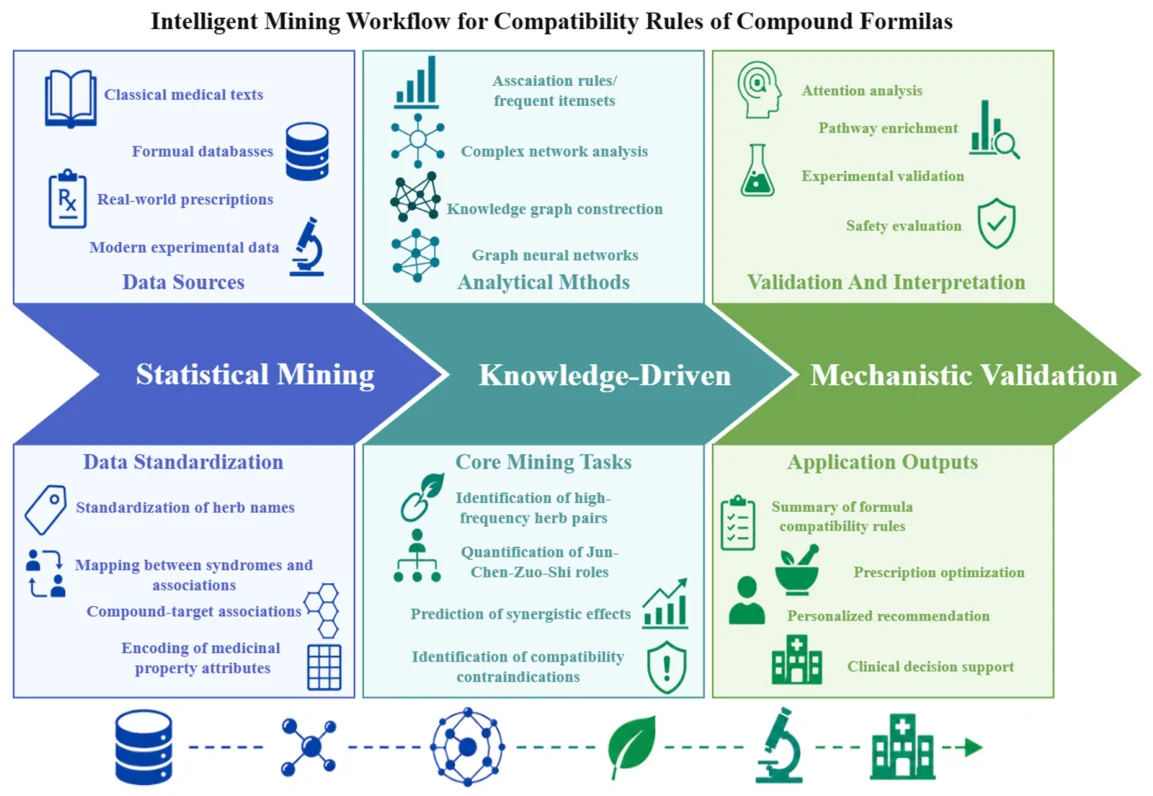
Workflow for identifying and evaluating formula-compatibility patterns. Statistical mining and graph models identify recurrent herb combinations and generate hypotheses about compatibility roles. Pathway analysis and attention weights support interpretation but do not establish pharmacological interaction. Experimental testing and safety assessment are required before any prescription or clinical implication is considered.

For prescription-oriented generation, predictive fit and compliance with safety constraints are separate objectives. Candidate safeguards include constraint-aware training, inference-time checks, and explicit representation of compatibility and dosage rules. These are design options whose effectiveness must be evaluated for the intended population and use [95,96]; the compatibility-analysis workflow is summarized in Figure 3.

### 3.6. Case Study I: GraphAI-for-TCM—Model Evaluation and Chemical Confirmation

#### 3.6.1. Research Background and Core Challenges

GraphAI was selected as Case Study I because it directly applies machine learning to TCM formula data, publishes reusable resources, and links compatibility representations to compound identification [74]. It is therefore classified as Tier I. Under the evidence hierarchy in Supplementary Table S1, its highest validation level is chemical confirmation by mass spectrometry. The model-derived targets, compatibility roles, and pathway links remain computational predictions that require experimental testing.

#### 3.6.2. Construction of the TCM-MKG Multidimensional Knowledge Graph

TCM-MKG contains 123,647 unique molecules identified by InChIKey in its chemical-compound module [74]. The module integrates compounds associated with decoction pieces and patent medicines together with compounds from broader drug–target resources, including DrugBank and TTD (primary Methods, Section 2.5 of the original study [74]). The neighbor-diffusion analysis instead uses 62,830 TCM-derived compounds (Section 3.2 of the original study [74]). These counts describe different scopes; the aggregate report does not provide a record-level crosswalk assigning all 60,817 additional records to specific categories. The graph also contains 226,589 decoction-piece–compound associations within an overall integration of 31 databases. Independent corroboration of individual edges could not be determined from the aggregate data. Database multiplicity therefore cannot be equated with independent corroboration of each association (Supplementary Table S5).

#### 3.6.3. Neighbor-Diffusion Strategy for Alleviating Compound–Target Sparsity

Before neighbor diffusion, 7631 of 62,830 TCM-derived compounds had recorded target annotations; afterward, 62,744 carried recorded or predicted annotations [74]. These counts yield coverage estimates of 12.145% and 99.863%, respectively (calculations in this review), rather than the source text’s 12.0% and 98.7%. The retained association set expanded from 343,373 recorded links to 6,526,940 links after a predicted pK_i_ > 5 filter (predicted K_i_ < 10 μM). The 6,183,567 computationally added links account for 94.74% of the retained set. The remaining recorded links should not all be classified as direct binding measurements without provenance-level inspection. Supplementary Table S5 preserves the source counts, reported percentages, and calculation rules separately.

#### 3.6.4. Graph Neural Network Architecture and Property Virtual Nodes

GraphAI represents each formula as a graph in which decoction-piece nodes carry 91-dimensional features for source attributes, medicinal properties, compatibility labels, therapeutic annotations, and dose [74]. Virtual nodes encode categorical properties such as Four Qi, Five Flavors, and meridian tropism. Message passing updates these representations and attention weights rank model-learned relationships among herbs. The architecture can quantify recurrent compatibility patterns in the training data; it does not demonstrate that highly weighted herbs are pharmacologically superior or that a ranked pair produces a beneficial biological interaction.

#### 3.6.5. Model-Derived Jun-Chen-Zuo-Shi Roles via Attention Weights

The graph-attention model maps the Jun-Chen-Zuo-Shi framework onto a directed weighted graph [74]. High outgoing or incoming attention is used to assign model-derived compatibility roles. These assignments summarize learned associations within the formula dataset. They do not independently demonstrate pharmacological hierarchy, interaction, or causal contribution. The reported herb rankings should therefore be interpreted as hypotheses for subsequent constituent-level and intervention-based testing.

#### 3.6.6. Formula Classification and Chemical Detection in Selected Herbs

GraphAI analyzed 215 COVID-19 prevention and treatment formulae collected from provincial and national guidance documents [74]. The model assigned most formulae to one TCM etiological category and ranked herb-pair attention patterns. UPLC-QToF-MS chemically detected 215 compounds in Astragali Radix and 17 in Phragmitis Rhizoma. Target and pathway links were then generated computationally using affinity thresholds. These results support a hypothesis that selected constituents may interact with neuroactive and xenobiotic-response pathways. They do not establish herb-pair synergy, clinical efficacy, or benefit in long COVID-19, all of which require direct experimental and clinical evaluation.

#### 3.6.7. Methodological Contributions and Limitations

**Figure 4:**
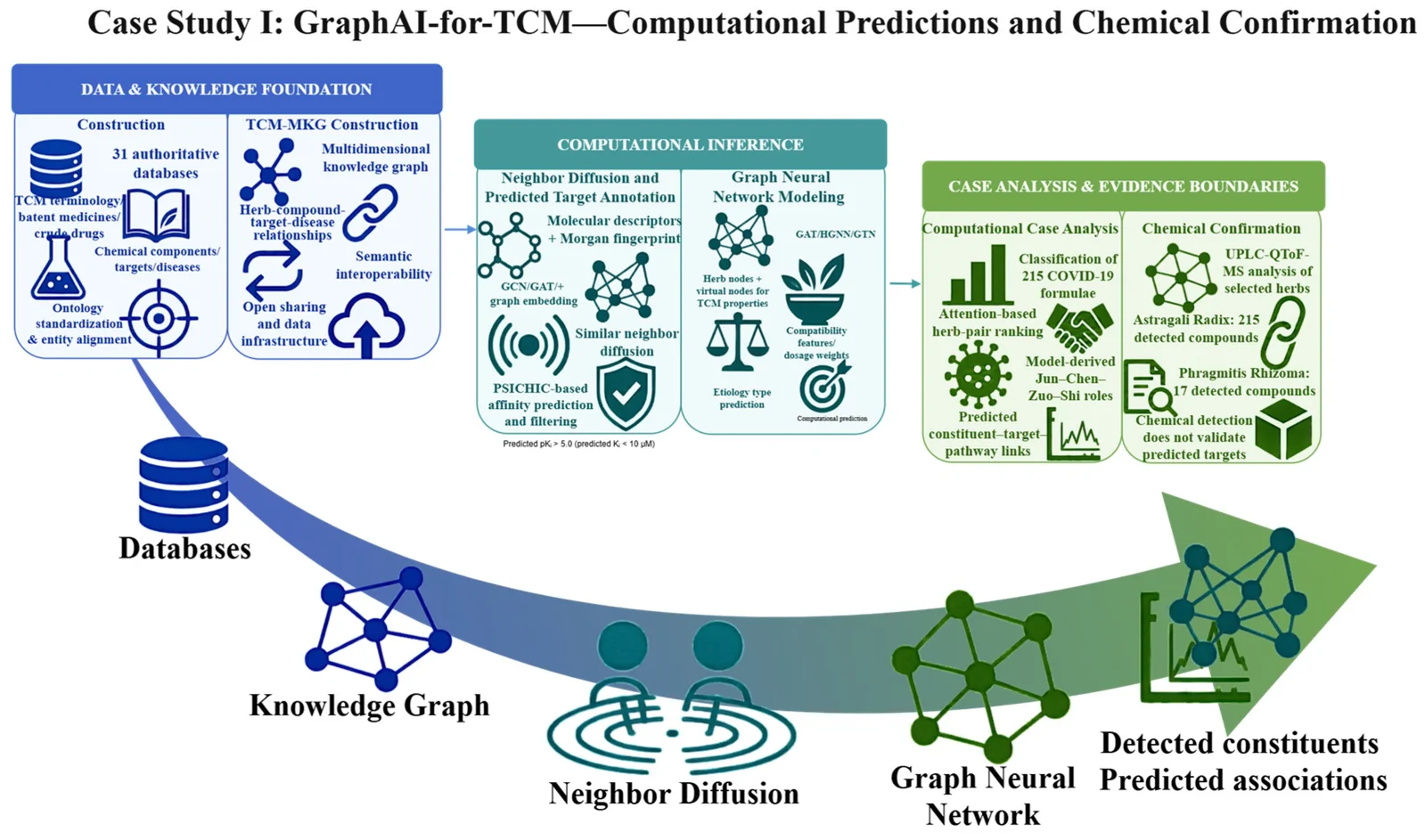
GraphAI-for-TCM and its evidence boundaries [74]. Database integration, neighbor diffusion, predicted-affinity filtering, and graph learning generate associations and formula classifications. UPLC-QToF-MS provides chemical confirmation in selected herbs. Model evaluation, chemical detection, and experimentally demonstrated target engagement are distinct forms of evidence; the predicted targets and pathways remain hypotheses.

GraphAI integrates a knowledge graph, graph neural networks, and attention-based interpretation within a reusable Tier I platform [74]. Its ontology is designed to improve semantic alignment across source databases, while attention weights provide comparable computational summaries of formula patterns. The neighbor-diffusion procedure reduces missing target annotations by generating predicted links. However, most conclusions remain computational, and mass spectrometry confirms constituents rather than predicted targets. The model also omits metabolic transformation, target-expression dynamics, host context, and direct clinical validation. Moreover, the available aggregate data do not permit calculation of the independently corroborated edge fraction (Section 3.6.2; Supplementary Table S5). Accordingly, its outputs are best treated as prioritization and hypothesis-generation tools. Figure 4 summarizes the GraphAI-for-TCM workflow and its evidence boundaries.

## 4. Multi-Omics Integration and Pharmacodynamic Associations

### 4.1. Metabolomics-Driven Characterization of Pharmacodynamic Associations

Metabolomics characterizes endogenous biochemical responses to herbal exposure [97,98,99,100,101]. Ma et al. studied Baiji Wuweizi Granule (BWG), a four-herb formula, in alcoholic liver injury [102]. Random forests selected serum metabolic markers [103], AdaBoost predicted compound–target relations, and shortest-path analysis connected the resulting networks. The study also included animal and protein-level measurements. These observations support activity in the tested model and selected molecular associations, while network paths, docking, and dynamics do not independently demonstrate direct binding or constituent-resolved causality.

Microbiota-depletion experiments provide stronger evidence than unconstrained correlation because they perturb a proposed intermediate component. In the cited Rhei Radix et Rhizoma study, attenuation of selected protective outcomes after antibiotic treatment supports microbiota dependence within that animal model [104]. The result does not identify a unique microbial taxon, metabolite, constituent, or human treatment effect. Multi-omics reproducibility also depends on instrument drift, batch effects, ion suppression, sample handling, and multiple-testing control [105]; these factors should be reported before pathway-level interpretations are advanced.

### 4.2. Multi-Omics Data Integration Frameworks

Multi-omics integration is highly dependent on task, data structure, and missingness. Cantini et al. compared nine joint dimensionality-reduction methods and found that performance varied across cancer datasets and analytical objectives [106]. Most methods required paired samples across omics layers, whereas approaches such as MOFA could accommodate partial missingness. These benchmarks support method selection within defined settings, not a universal ranking. Key risks include heterogeneous measurement scales, high-dimensional small-sample regimes, weak cross-modal correspondence, and batch effects.

Multi-omics integration is a multi-view learning problem: each measured layer provides a distinct, potentially complementary view of the biological system [107,108]. Models differ in how they encode shared factors, cross-view dependence, supervision, and missing observations [106]. Genomic, transcriptomic, proteomic, and metabolic processes can also be connected by directional regulatory cascades and feedback. Incorporating such relationships as priors may guide interpretation, but an inferred latent factor or cross-layer association is not a demonstrated causal cascade. Directionality requires temporal information, perturbation, or other defensible identification assumptions.

The NP-AI-omics framework links network pharmacology, artificial intelligence, and multi-omics within a proposed discovery workflow [18]. Microbial and metabolic measurements can extend this framework [109], but proposed biological mediators require direct temporal or intervention-based testing. Method-specific capabilities and limitations are summarized in Supplementary Table S6.

**Figure 5:**
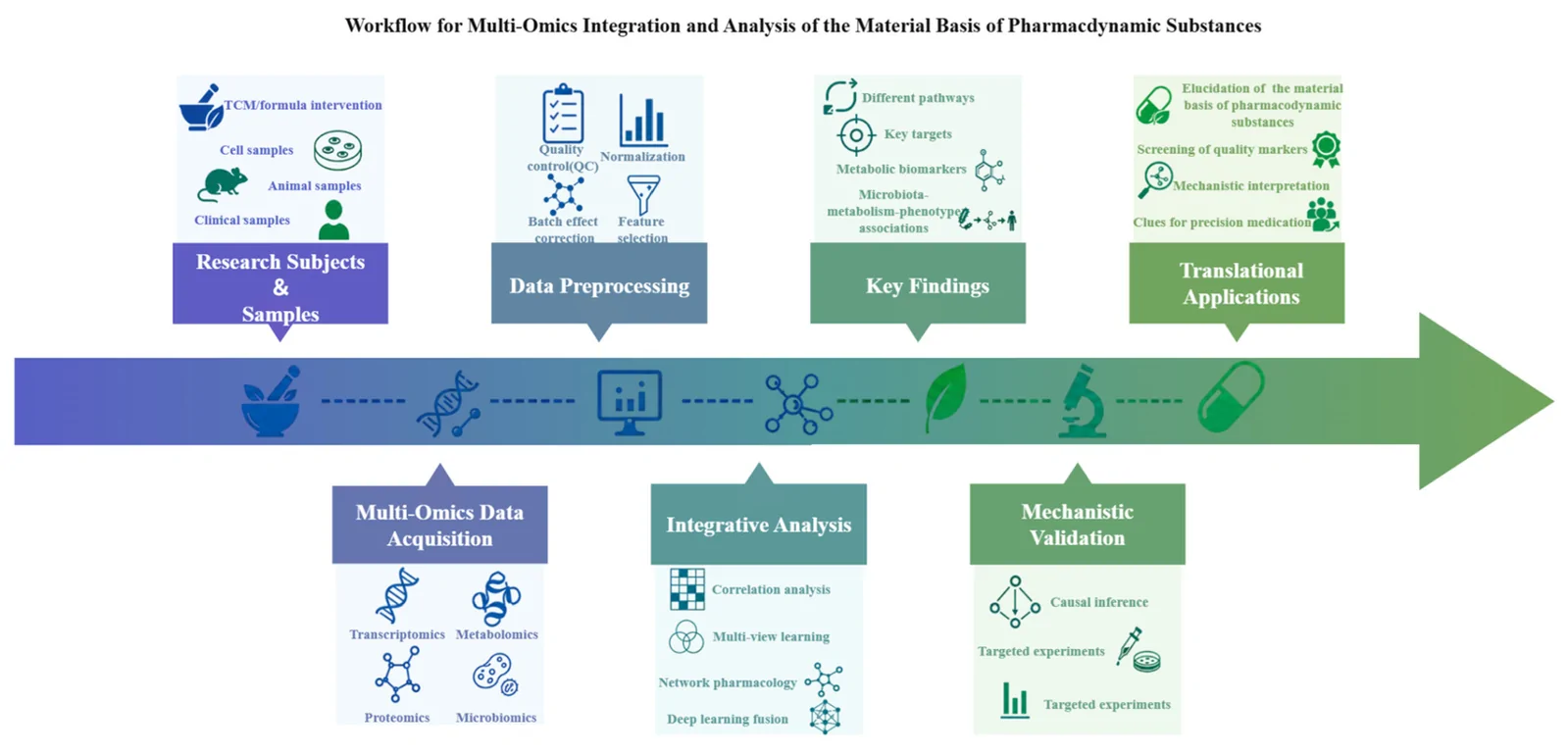
Multi-omics workflow for evaluating pharmacodynamic material evidence. Samples undergo quality control, normalization, batch assessment, and feature selection before integration. The resulting associations can prioritize metabolites, targets, and pathways. Causal or clinical conclusions require additional intervention-based or human evidence and are not implied by multi-omics integration alone.

Multi-omics evidence can refine computational hypotheses when sample pairing, batch effects, missingness, and measurement uncertainty are addressed. Quality-marker and gut–microbiome applications are relevant here when they contribute to constituent identification, activity assessment, or validation [109,110,111,112,113,114,115,116,117,118]. Figure 5 summarizes the analytical sequence; Supplementary Table S1 distinguishes application scope from the evidence actually obtained.

### 4.3. Case Study II: A Two-Stage “Untargeted–Targeted” Metabolomics Validation Framework for Huo-Luo-Xiao-Ling-Dan (HLXLD) in Alzheimer’s Disease

#### 4.3.1. Research Design and Scientific Rationale

Case Study II examines HLXLD in an Alzheimer-like rat model [119]. It is classified as Tier I because it evaluates a TCM formula, and its highest evidence level is animal validation. The study combines untargeted and targeted metabolomics with behavioral and histopathological measurements. It does not include a machine learning screening step or identify active compounds through a predictive model. We retain it as a boundary case showing what downstream experimental validation can contribute after candidate prioritization, while explicitly separating it from the machine learning cases.

#### 4.3.2. Animal Modeling and Pharmacodynamic Confirmation

The study evaluated HLXLD in an AlCl_3_/D-galactose-induced rat model using the Morris water maze and hippocampal histopathology [119]. The reported improvements support animal-level pharmacodynamic activity. The model and these outcomes do not establish efficacy in human Alzheimer disease.

#### 4.3.3. Untargeted Metabolomics: Panoramic Discovery in Brain Tissue

Untargeted UHPLC-Q-Exactive-Orbitrap-MS analysis of brain tissue, followed by multivariate statistics, identified 32 altered metabolites: 26 decreased and six increased in the reported comparison [119]. The associated pathways included amino acid, tricarboxylic acid, arachidonic acid, and lipid metabolism. This discovery stage detects metabolic responses; it does not identify the formula constituents responsible for them.

#### 4.3.4. Targeted Metabolomics: Multidimensional Quantitative Validation

Targeted UHPLC-QQQ-MS extended the measurements to brain and plasma [119]. Compared with the model group, reported changes involved 8/21/7/16 analytes in brain and 9/20/6/2 in plasma for neurotransmitters, amino acids, arachidonic-acid-related metabolites, and lipids, respectively. These category counts describe endogenous metabolic responses, not numbers of active herbal constituents or proven targets.

#### 4.3.5. Integrating Metabolic Associations into a Functional Interpretation

The two metabolomic stages identified coordinated changes in neurotransmitter, energy, lipid, and arachidonic acid pathways [119]. These measurements support associations between HLXLD exposure and metabolic recovery in the animal model. They do not identify which individual constituents produced each effect or establish a causal molecular pathway. The results are therefore presented as an animal-level pharmacodynamic pattern that requires constituent-resolved and intervention-based follow-up.

#### 4.3.6. Methodological Contributions and Limitations

**Figure 6:**
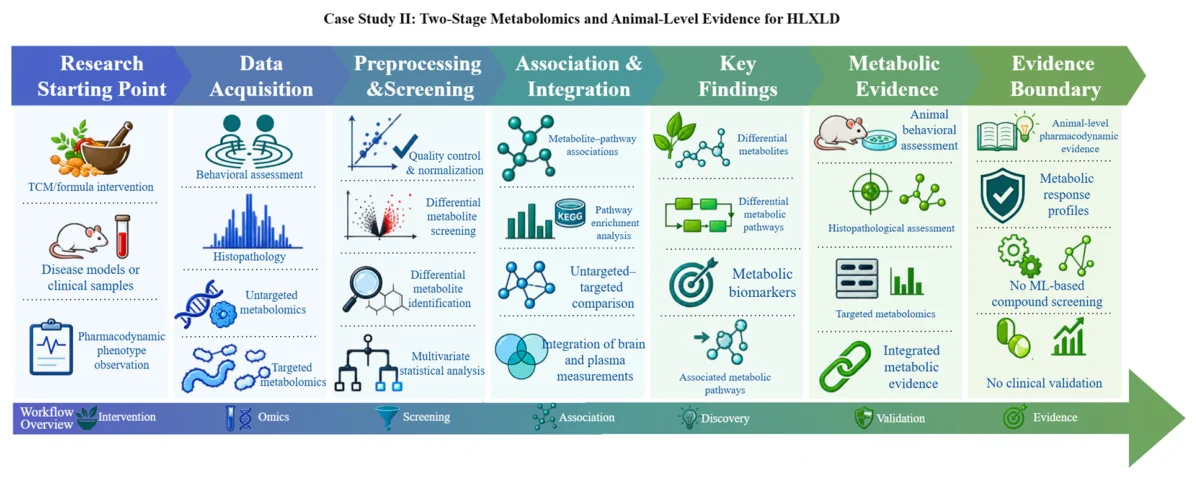
Actual evidence sequence in Case Study II. HLXLD was evaluated in an Alzheimer-like rat model using behavioral testing and histopathology, followed by untargeted metabolomics for broad discovery and targeted metabolomics for quantitative follow-up [119]. The study did not perform machine learning compound screening, target prediction, microbiome analysis, or clinical translation.

The untargeted-to-targeted sequence provides discovery followed by quantitative analytical follow-up [119]. Its interpretation remains limited by an induced animal model, unresolved constituent attribution, and the need to verify analytical controls in the full protocol. As the accessible report did not specify whether multiplicity correction was applied (a limitation in reporting and verification rather than evidence that no such correction was performed), the statistical robustness of the findings could not be assessed. This case contributes downstream animal and metabolic evidence, rather than an end-to-end machine learning screening demonstration. Figure 6 summarizes the experimental sequence and the animal-level evidence obtained in this case study.

## 5. Drug Development and Repurposing

### 5.1. Database-Enabled Drug Repurposing

Database-enabled discovery depends on the scope and provenance of the underlying resources. TCMSP, HERB 2.0, ETCM, TCMM, TTD, DrugBank, and PubChem provide complementary chemical, target, clinical, and pharmacological records [75,76,120,121,122,123,124]. Applications that use these resources for compound or target prioritization are Tier I when the input and endpoint are TCM-specific. Image recognition and non-destructive quality-control datasets are treated as contextual applications unless they connect material identity to a verified activity endpoint [125,126].

For any database-derived result, the analytical record should preserve source-level provenance and unresolved mapping decisions. Reports should specify database versions and access dates, identifier harmonization, preprocessing, deduplication, and the number of records excluded at each step. If multiple databases cover the same entities, the main conclusion should be tested for cross-database stability. A finding that changes with the selected database should be reported as database-dependent rather than biologically robust [19,127,128].

### 5.2. Generative Modeling for Natural Product-Inspired Molecular Design

Generative models have shown value in general molecular-design settings [129,130,131,132], but most examples remain Tier III for TCM. NIMO was evaluated on structure-guided, activity-oriented, and pocket-conditioned tasks using natural-product-related training sources [132]. Other cited candidates advanced through experimental or clinical development outside TCM [129,131,133,134]. These studies demonstrate technical feasibility within their original settings, not validated clinical utility for TCM-derived therapeutics.

Natural products challenge generative models because of their stereochemical density, ring complexity, and three-dimensional structure. Two feasibility concepts should be evaluated separately. Synthetic accessibility is the practical attainability of a candidate through laboratory chemical operations, considering precursor availability, step count, reaction compatibility, stereochemical control, purification, scale, and safety. Biosynthetic feasibility is the plausibility that a native or engineered biological system can produce the structure through an enzyme-supported pathway with suitable substrates and host context. A compound can be difficult biosynthetically yet obtainable chemically, or the reverse. Candidate acquisition may proceed by total chemical synthesis, semisynthesis from an isolated natural scaffold, whole-cell or enzyme-mediated biotransformation, or a chemoenzymatic sequence [63,64,65,129,130]. These assessments are route-specific hypotheses until experimentally demonstrated.

For TCM-related generation, candidates should be evaluated separately for natural-product relevance, route feasibility, and measured activity. Supplementary Table S7 distinguishes source-supported model descriptions from route-specific evaluation requirements. A chemical-validity score or a general drug-discovery benchmark cannot establish that a generated TCM candidate can be produced or is pharmacologically active.

### 5.3. Toxicity Prediction and Safety Assessment

Safety models must be interpreted within their evaluation design [135,136,137]. The DILI-ML study reported a mean accuracy of 0.631 under ten-fold cross-validation and evaluated failed drug candidates externally [138]. ToxPredictor used a 300-compound transcriptomic resource, trained on 163 compounds with unambiguous labels, and reserved 51 compounds for a fully independent blind experiment. The reported sensitivity and specificity were calculated from 47 compounds, comprising 33 DILI-positive and 14 DILI-negative compounds. The model correctly classified 29 of the 33 positives and all 14 negatives, yielding 88% sensitivity and 100% specificity [139]. HERGAI reported cross-validation recall for hERG inhibition and included an external test [140]. These results use different endpoints, class balances, thresholds, and validation sets. They therefore cannot be ranked directly or treated as evidence for complex in vivo toxicity in TCM. Supplementary Table S2 records the available context.

Machine learning currently supports candidate prioritization and selected safety endpoints, but complete prediction-to-experiment workflows remain uncommon in TCM. Benchmark accuracy, external validation, and clinical outcomes represent different evidence levels and should be reported separately. No cited study establishes that a machine learning-designed TCM-derived therapeutic has reached the market.

Supplementary Table S8 summarizes endpoint-specific toxicity evidence and validation boundaries. Predictive performance on one assay or injury label should not be extended to whole-organism or formula-level safety without additional testing.

### 5.4. Case Study III: Proposed Evidence Framework for the TCM–Gut–Brain–Microbiome Hypothesis

#### 5.4.1. Research Background and the Causal Problem

Case Study III examines how separate TCM, microbiome, metabolite, animal, and human observational studies can inform a testable evidence framework [18,104,109,141,142,143]. It is Tier I only for the constituent and animal studies directly involving TCM. The assembled gut–brain–microbiome chain has not been tested as a whole. Its highest constituent-specific validation level is animal evidence, while the human evidence is observational and derives from non-TCM cohorts. The case is therefore presented as a proposed hypothesis framework rather than a completed causal demonstration.

#### 5.4.2. Multi-Omics Evidence Components and Candidate Intermediate Variables

Separate studies identify neural, endocrine, metabolic, and immune routes through which microbiota-derived signals may be associated with host phenotypes [109]. High-fiber interventions and microbial metabolites provide experimental support within their original models [142], while human cohort studies associate microbial variation with quality-of-life and depression-related measures [141]. These findings do not test a TCM-derived compound-to-microbiota-to-brain chain in one design. They are therefore used here to define candidate intermediate variables and interventions for future studies, not to claim a demonstrated mediation pathway.

#### 5.4.3. TCM Evidence Component: Rhubarb Fractions in Acute Kidney Injury

Gao et al. compared rhubarb total extract, an anthraquinone-rich fraction, a tannin fraction, and the remaining fraction in rats [143]. Oral pretreatment for five consecutive days preceded HgCl_2_-induced kidney injury. The total extract and anthraquinone-rich fraction reduced renal injury indicators, including serum creatinine and blood urea nitrogen, and LC-MS characterized the fraction’s anthraquinones. This supports preventive, fraction-level activity in that animal model. It does not establish efficacy of each isolated constituent, microbiota mediation, or a gut–brain mechanism. Its connection to the other studies in Case Study III is therefore hypothesis-generating.

#### 5.4.4. The Causal Inference Toolkit: Methodological Integration from Correlation to Causation

Moving from association to causal inference requires a structured set of analyses and interventions. Statistical mediation can estimate indirect associations, but uncontrolled confounding limits causal interpretation [144,145]. Antibiotic depletion, fecal microbiota transplantation, and germ-free models can test whether microbial changes are necessary or sufficient within a defined experimental system [109]. Bradford Hill considerations can organize evidence across temporality, consistency, intervention, and plausibility [146]. None of these elements alone validates the assembled TCM–gut–brain–microbiome chain. A credible test would require prospectively linked computational predictions, multi-omics measurements, and targeted interventions [18,147].

#### 5.4.5. Methodological Contributions and Implications for the Field

**Figure 7:**
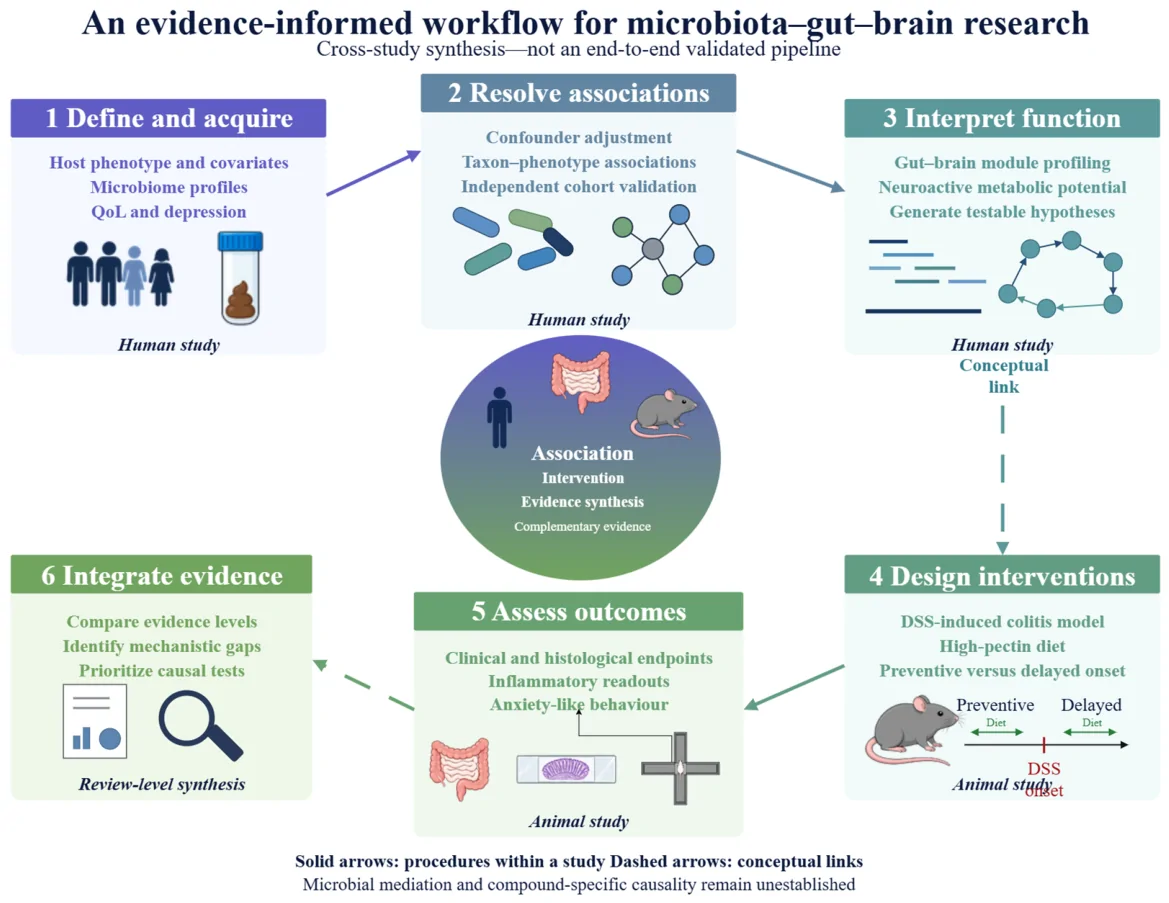
Cross-study evidence components and proposed links for Case Study III. The Rhei Radix traumatic-brain-injury study [104] and rhubarb-fraction acute-kidney-injury study [143] provide distinct TCM animal evidence. Dietary-fiber and acetate experiments in mouse colitis [142] and a non-TCM human observational cohort [141] supply separate context. Dashed cross-study links denote hypotheses requiring direct tests; the assembled sequence has not been demonstrated in one study.

The proposed framework yields three bounded recommendations. First, multi-omics associations should be paired with interventions that test the proposed mediator. Second, dosing schedule and baseline microbiota should be specified because intervention timing can alter observed effects [142]. Third, animal findings require independent human evaluation, and human associations do not establish treatment effects [141]. The framework remains limited by unmeasured constituent metabolism, nonlinear dose–microbiota relationships, and functional redundancy among microbial taxa. These limitations define experiments needed to test the hypothesis rather than mechanisms already established. Figure 7 summarizes the cross-study evidence components and the proposed links that require further testing.

## 6. Challenges and Future Research Directions

Three limitations recur across the reviewed evidence. First, models trained on synthetic compounds may not generalize to structurally distinct natural products. Second, model explanations and TCM concepts often remain associations rather than causal accounts. Third, database inconsistency, missing negative results, and uneven validation interrupt the progression from prediction to reproducible experimental evidence. Future work should therefore prioritize applicability-domain testing, explicit uncertainty, traceable database provenance, and intervention-based validation.

## 7. Conclusions

Machine learning can currently support candidate prioritization, relation mining, and selected property or safety predictions when the training domain and evaluation design are stated clearly. These uses are most reliable as decision-support tools, not substitutes for chemical and experimental confirmation.

The evidence remains insufficient for broad claims of mechanism, formula synergy, clinical efficacy, or clinical utility. Many TCM examples stop at computational association, while several influential benchmarks were developed outside natural-product chemical space. Database dependence and limited reporting of negative results further constrain reproducibility.

Clinical progression requires independent prospective validation, chemical and target confirmation, and staged animal and human evidence. Claims should remain proportionate to the evidence available.

### Minimum Reporting Checklist

For each item in Table 3, provide the requested information or explicitly state “not applicable” or “not reported,” with a reason. The checklist defines reporting requirements; it does not imply that every study must include a knowledge graph or complete every experimental stage. Supplementary Table S9 provides detailed diagnostics for split integrity.

## Figures and Tables

**Table 1:** Molecular representations and their interpretation boundaries. Rows describe capabilities and evaluation conditions, not an ordinal ranking of accuracy, cost, or natural-product suitability. Quantitative benchmark context is provided in Supplementary Table S2.

Representation/Examples	Input and Encoded Information	Evaluation/Interpretation Boundary	Sources
ECFP/Morgan + descriptor models	Atom-centered substructures or defined physicochemical descriptors; usable with linear models, RF, or SVM.	Report radius, vector size, hashing, endpoint, and split. Suitability for natural products is dataset-dependent.	[20,21,22,23,24,25]
Molecular graph networks	Atom/bond graphs; learned local neighborhoods and graph-level features.	Architectural detail and data coverage determine performance; topology alone does not establish mechanism.	[20,23,32]
GROVER	Self-supervised molecular graph representations; 10 million molecules in the reported pretraining corpus.	General molecular benchmarks; task-specific results are not evidence of TCM transfer.	[29]; Table S2
Chemical language models	Molecular strings and their contextual patterns; examples include MolFormer-XL.	Equivalent strings, stereochemistry, conformations, and chemical-domain shift require explicit handling.	[30,32,33,54]
Uni-Mol	Three-dimensional molecular conformations and spatial relationships.	The docking benchmark tests binding-pose prediction, not TCM efficacy or target engagement.	[27]; Table S2
Q-GEM	Geometry enhanced with quantum-chemical information.	Benchmark gains depend on the particular task and comparator; no universal natural-product suitability grade.	[28]
CRA	Contextual molecular representations for few-shot prediction.	Selection bias and support/query protocols constrain interpretation; cited results use the specified preprint version.	[31]; Table S2

**Table 2:** Representative network resources and methods relevant to formula analysis. Functional descriptions are source-based; interpretation boundaries are the present review’s synthesis. Scope depends on the data and application, rather than on a universal tier assigned to an algorithm.

Resource/Method	Type and Function	Use and Evidence Boundary	Sources
Cytoscape/ClueGO	Network visualization and functional annotation software. These tools are reviewed as resources; no version-specific analysis was performed in this review.	Displays or groups supplied relationships; visualization and enrichment are not independent validation.	[78,79]
STRING	Protein-association database and analysis resource.	Distinguish evidence channels and confidence settings; protein association need not represent direct physical binding.	[80]
Pathway enrichment workflows/Metascape	Functional enrichment and systems-level annotation.	Report gene universe, annotation version, statistical test, and multiple-testing adjustment.	[81,82]
BATMAN-TCM 2.0	TCM ingredient–target resource containing known and predicted links.	Retain link provenance and distinguish curated records from computational predictions.	[83]
TCMM/ETCM	TCM data integration resources for formulae, herbs, ingredients, targets, and diseases.	Entity types and release snapshots differ; harmonization is needed before counts are compared.	[75,76]
OpenTCM	Text-derived TCM knowledge graph and retrieval framework.	Textual relations and generated retrieval outputs need source verification, not chemical or clinical confirmation.	[73]
HTINet2	Knowledge-graph and graph-network herb–target ranking.	Reported random relation tests do not establish unseen-herb or independent-database generalization.	[77]
GraphAI-for-TCM	Knowledge graph, neighbor diffusion, and graph-attention formula analysis.	Separate model performance, constituent detection, and predicted target edges; attention is an association summary.	[74]; Table S5
NIMS	Network-based prioritization of potentially synergistic combinations.	Experimental support is restricted to tested combinations and endpoints in the original study.	[68]

**Table 3:** Minimum reporting checklist for machine learning studies relevant to TCM bioactive-compound discovery.

Reporting Item	Minimum Information
1. Scope and endpoint	Define TCM/NP/general scope, endpoint, independent sample unit, sample size, and class balance.
2. Data provenance	Report database versions, access dates, identifiers, mapping rules, exclusions, and deduplication.
3. Graph evidence	Count predicted additions, pre-existing records, and directly measured links separately; define each category.
4. Denominators	Specify each counted universe, subset, unit, and arithmetic rule; explain differing coverage totals.
5. Independent support	Report per-edge underlying sources where available; distinguish independent evidence from duplicate database imports.
6. Split integrity	Split before learned preprocessing; group repeated entities and preserve a fully held-out test set.
7. Similarity overlap	State representation, metric, threshold, and rationale; give cross-split exact/near-duplicate counts and disposition.
8. Extrapolation	Use scaffold, grouped, temporal, external, or prospective tests consistent with the intended generalization claim.
9. Model comparison	Use matched endpoints, partitions, tuning budgets, metrics, thresholds, and uncertainty reporting.
10. Applicability domain	Report domain shift, predictive uncertainty, abstention rules, and coverage–performance trade-offs.
11. Robustness and failures	Report cross-database sensitivity, negative results, failed predictions, and relevant analytical controls.
12. Evidence and causal links	State the highest stage actually reached, constituent attribution, and untested links assembled across studies.

## Data Availability

No new data were created or analyzed in this study. Data sharing is not applicable to this article.

## Supplementary Materials

The following supporting information can be downloaded at: https://www.mdpi.com/article/10.3390/ph19101567/s1; Table S1. Application scope and evidence hierarchy. Table S2. Dataset and validation context for quantitative claims. Table S3. Bibliographic record traceability. Table S4. Database snapshots, entity definitions, and counting rules. Table S5. GraphAI counts, denominators, and evidence provenance. Table S6. Multi-omics method capabilities and selection conditions. Table S7. Generative-model and acquisition-route evidence. Table S8. Endpoint-specific toxicity-prediction evidence. Table S9. Validation, leakage, and provenance reporting checklist. Table S10. Source context for selected numerical claims. Table S11. Comparison with recent TCM-AI reviews. References [148,149,150,151,152,153,154,155,156,157,158] are cited in the supplementary materials.

## Abbreviations

The following abbreviations are used in this manuscript:

AD	Alzheimer’s disease
ADME	Absorption, distribution, metabolism, and excretion
ADMET	Absorption, distribution, metabolism, excretion, and toxicity
AI	Artificial intelligence
AUC	Area under the curve
CNN	Convolutional neural network
CPM	Chinese patent medicines
CRA	Contextual Representation Anchor
DILI	Drug-induced liver injury
DOID	Disease Ontology identifier
ECFP	Extended-connectivity fingerprint
FMT	Fecal microbiota transplantation
Fsp^3^	Fraction of sp^3^-hybridized carbons
GAE	Graph autoencoder
GAT	Graph attention network
GBM	Gut–brain–microbiome
GCN	Graph convolutional network
GNN	Graph neural network
GTN	Graph transformer
H&E	Hematoxylin and eosin
HDLSS	High-dimension low-sample-size
hERG	Human ether-à-go-go-related gene
HGNN	Hypergraph neural network
HLXLD	Huo-Luo-Xiao-Ling-Dan
HR	Hit rate
ICD-11	International Classification of Diseases, 11th Revision
InChI	International Chemical Identifier
KGE	Knowledge graph embedding
MeSH	Medical Subject Headings
MPNN	Message passing neural network
MRM	Multiple reaction monitoring
NDCG	Normalized discounted cumulative gain
NIMS	Network-based Identification of Multicomponent Synergy
OOD	Out-of-distribution
PAINS	Pan-assay interference compounds
PBPK	Physiologically based pharmacokinetic
PO	Botanical origins
PPI	Protein–protein interaction
PSICHIC	Physicochemical graph neural network for protein–ligand interaction
QSAR	Quantitative structure–activity relationship
Q-marker	Quality marker
RF	Random forest
SCFA	Short-chain fatty acid
SHAP	SHapley Additive exPlanations
SMILES	Simplified molecular-input line-entry system
SVM	Support vector machine
TBI	Traumatic brain injury
TCM	Traditional Chinese medicine
TCM-MKG	Multidimensional TCM Knowledge Graph
TTI	TCM ingredient–target interaction
UMLS	Unified Medical Language System
XAI	Explainable artificial intelligence
