# Supplementary material for: Machine Learning for Screening Bioactive Compounds in Traditional Chinese Medicine: Computational Prediction, Multi-Omics Integration, and Clinical Translation Potential
Source: Pharmaceuticals (Basel). 2026 Oct 2;19(10):1567. doi: 10.3390/ph19101567 (PMC13708095; doi:10.3390/ph19101567)
Supplement: Supplementary file 1 [file pharmaceuticals-19-01567-s001.zip › pharmaceuticals-4553587-supplementary.pdf]

Supplementary Materials

Machine Learning for Screening Bioactive Compounds in Traditional Chinese Medicine: Computational Prediction, Multi-Omics Integration, and Clinical Translation Potential

These tables distinguish application scope, evidence stage, and evaluation context. A blank evidence cell means that the stage is not established by the information summarized here; it is not proof that the study did not perform the procedure. Bibliographic record checks were updated on 11 September 2026. Retained database-snapshot notes in Table S4 are dated 7 September 2026 in the supplied materials. Entries 26 and 33 in Table S3 were updated on 30 September 2026 to align with the main manuscript. High-resolution versions of main-text Figures 1, 2, 4, 5, 6, and 7 are provided after Table S11.

Table S1. Application scope and evidence hierarchy

Scope tiers and evidence levels answer different questions. Scope indicates where a method was applied. Evidence level indicates the highest validation stage reached. A higher scope tier number does not imply stronger evidence.

Tier	Meaning	Decision rule
Tier I	Direct TCM application	TCM formulae, constituents, interventions, databases, or endpoints are analyzed directly.
Tier II	Natural-product or herbal validation	The method is tested in natural-product or herbal settings but not in a TCM-specific screening task.
Tier III	General or prospective relevance	The method is evaluated in general drug-discovery settings and remains prospective for TCM.

Level	Evidence stage	Operational definition
1	Computational prediction	In silico ranking, classification, docking, network inference, or simulated property prediction.
2	Chemical confirmation	A constituent or structure is confirmed by an analytical chemistry method such as LC-MS.
3	Biochemical testing	Direct activity, binding, enzyme, or target-engagement testing in a cell-free system.
4	Cellular validation	Activity or mechanism-related endpoints are tested in cells or ex vivo biological systems.
5	Animal study	Efficacy, pharmacology, toxicity, or mediation is tested in an animal model.
6	Human observation	Associations or biomarkers are evaluated in human cohorts without interventional treatment evidence.
7	Clinical evidence	A therapeutic claim is evaluated in a clinical trial or other treatment-effect design in humans.

The highest stage reached does not imply that all preceding stages were completed. In particular, animal evidence for a formula does not establish the mechanism or activity of each isolated constituent.

Example	Ref.	Scope	Prediction	Chemical	Biochemical	Cell	Animal	Human obs.	Clinical	Highest level	Boundary
GraphAI-for-TCM	[74]	I	Yes	Yes						Chemical confirmation	MS confirmed constituents; targets and pathways remained predicted.
HLXLD metabolomics	[119]	I	Analytical statistics	Yes			Yes			Animal study	Animal behavior, histology, and metabolomics; no ML screening step.
Rhei Radix TBI study	[104]	I		Yes			Yes			Animal study	Microbiota depletion provided animal-level intervention evidence.
Anthraquinone AKI study	[143]	I		Yes			Yes			Animal study	Five-day oral pretreatment before $HgCl_2$ injury; rhubarb extract/fraction-level animal protection and LC-MS characterization [143]; no microbiota or gut–brain causal test.
HTINet2	[77]	I	Yes							Computational prediction	Held-out relation split; no external biological validation reported here.
Uni-Mol docking	[27]	III	Yes							Computational prediction	General docking benchmark, not TCM or natural-product validation.
SSnet	[62]	III	Yes							Computational prediction	Experimental databases supplied labels; the model output remained computational.
ToxPredictor	[139]	III	Yes			Yes				Cellular validation	Primary human hepatocyte transcriptomics with independent blind compound evaluation.
Valles-Colomer cohort	[141]	III	Computational analysis					Yes		Human observation	Human microbiome associations; not a TCM intervention study.
AI-discovered drugs in trials	[134]	III	Yes						Yes	Clinical evidence	General AI drug-development evidence; no TCM-derived therapeutic identified.
BWG alcoholic liver injury	[102]	I	Yes	Yes			Yes			Animal study	Serum-marker RF, compound–target AdaBoost, animal and protein measurements; network paths and docking are not direct binding confirmation.

Table S2. Dataset and validation context for quantitative claims

Results are study-specific and should not be ranked across rows. Reporting limits distinguish information absent from a cited table or abstract from an analysis shown not to have been performed.

Model	Ref.	Task	Training data	Test or split	Validation type	Reported result	Comparator	Interpretation limit
Uni-Mol docking	[27]	Binding-pose prediction	PDBbind General Set v2020 after similarity exclusion	CASF-2016, 285 complexes	Held-out external benchmark	70.53% below 1 Å; 88.07% below 2 Å	AutoDock Vina: 44.21% / 64.56% at exhaustiveness 8	ICLR 2023 / cited supplementary benchmark table; no confidence intervals accompany these percentages in that table. Not TCM/NP validation.
GROVER	[29] (primary NeurIPS 2020 paper)	11 MoleculeNet property tasks	10 million unlabeled molecules for pretraining	Scaffold-balanced task splits; three folds in released evaluation	Within-benchmark evaluation	Task-specific results on 11 MoleculeNet tasks; no pooled improvement used here.	Study-specific baselines	Endpoints and metrics differ; performance must be interpreted against each task's comparator and scaffold-balanced split.
CRA	[31]	Few-shot molecular property prediction	MoleculeNet and FS-Mol protocols	Benchmark-specific support/query tasks	Few-shot benchmark and domain-transfer evaluation	+2.60 percentage points in AUC	Study-specific state of the art	arXiv:2410.20711v2 (2024); few-shot benchmark evidence, not prospective TCM validation.
HTINet2	[77]	Herb-target ranking	38,002 known relationships	8:1:1 train/validation/test relation split	Held-out random relation test	HR@10 0.4458; NDCG@10 0.3746	Approximately +122.8% and +35.7% versus best baseline	Does not test unseen-database or unseen-herb transfer
SSnet	[62]	Protein-ligand interaction prediction and throughput	Experimental BindingDB plus study-specific datasets	BindingDB, DUD-E, human and C. elegans evaluations	Internal/benchmark tests; throughput on NVIDIA P100	1 million scores in about 18 minutes	Several docking and ML baselines	Throughput is not predictive or biological validation
GraphAI formula model	[74]	Five formula categories	4,256 of 6,080 formulae	Validation 1,222; test 602; five-fold CV for tuning	Held-out test after model selection	AUC 0.8918; accuracy 0.7924; F1 0.6790 for reported settings	GTN, HGNN, and GAT within one study	Formula classification does not validate predicted targets
DILI-ML	[138]	Drug-induced liver injury	Several thousand compounds	Ten-fold cross-validation plus failed-drug external evaluation	Internal CV and external candidate assessment	Mean accuracy 0.631	Study-specific models	Endpoint and class balance differ from other toxicity studies
ToxPredictor	[139]	Drug-induced liver injury from hepatocyte transcriptomics	300-compound resource; 163 labeled compounds used for training	Independent blind set of 51, including 33 DILI+, 14 DILI-, 4 unknown	Separate experiment withheld from model development	88% sensitivity; 100% specificity; 29/33 and 14/14	Matched preclinical comparators	Independent compound set; not a TCM-specific safety study
HERGAI	[140]	hERG blocker prediction	1,937 blockers and 297,990 non-blockers	Five-fold CV plus external test	Internal CV and external test	CV recall 0.931	Study-specific baselines	Class imbalance and threshold affect interpretation

Table S3. Bibliographic record traceability

This table covers all 147 numbered references. DOI registration matching establishes record identity only. Selected case mismatches, publication-year conflicts, and non-DOI records were checked against primary publisher, proceedings, or repository records. Version and online/issue-year differences are stated explicitly. Claim-level support is evaluated in the relevant case and evidence tables.

Ref.	Identifier / record	Bibliographic record	Check scope	Version / evidence boundary
1	DOI 10.1038/nrd4510	Harvey et al. (2015). The re-emergence of natural products for drug discovery in the genomics era	DOI registration record matched	Record traceability does not establish claim-level support.
2	DOI 10.1038/s41573-020-00114-z	Atanasov et al. (2021). Natural products in drug discovery: advances and opportunities	DOI registration record matched	Record traceability does not establish claim-level support.
3	DOI 10.1021/acs.jnatprod.9b01285	Newman et al. (2020). Natural Products as Sources of New Drugs over the Nearly Four Decades from 01/1981 to 09/2019	DOI registration record matched	Record traceability does not establish claim-level support.
4	DOI 10.1007/s10822-013-9672-4	Polishchuk et al. (2013). Estimation of the size of drug-like chemical space based on GDB-17 data	DOI registration record matched	Record traceability does not establish claim-level support.
5	DOI 10.1016/j.ijpharm.2025.125789	Jarallah et al. (2025). Artificial intelligence revolution in drug discovery: A paradigm shift in pharmaceutical innovation	DOI registration record matched	Record traceability does not establish claim-level support.
6	DOI 10.1016/j.canlet.2025.217821	Sarvepalli et al. (2025). Role of artificial intelligence in cancer drug discovery and development	DOI registration record matched	Record traceability does not establish claim-level support.
7	DOI 10.1038/nrd.2017.232	Schneider et al. (2018). Automating drug discovery	DOI registration record matched	Record traceability does not establish claim-level support.
8	DOI 10.1016/j.ibmed.2025.100316	Othman et al. (2025). Artificial intelligence for natural product drug discovery and development: current landscape, applications, and future directions	DOI registration record matched	Record traceability does not establish claim-level support.
9	DOI 10.1016/j.sbi.2023.102771	Cichońska et al. (2024). AI for targeted polypharmacology: The next frontier in drug discovery	DOI registration record matched	Record traceability does not establish claim-level support.
10	DOI 10.1038/s41573-023-00774-7	Mullowney et al. (2023). Artificial intelligence for natural product drug discovery	DOI registration record matched	Record traceability does not establish claim-level support.
11	DOI 10.1016/s1875-5364(25)60983-6	Yan et al. (2025). Artificial intelligence in traditional Chinese medicine: from systems biological mechanism discovery, real-world clinical evidence inference to personalized clinical decision support	DOI registration record matched	Record traceability does not establish claim-level support.
12	DOI 10.34133/research.1224	He et al. (2026). Artificial Intelligence in Traditional Chinese Medicine: Unraveling Herbal Medicine's Mechanisms	DOI registration record matched	Record traceability does not establish claim-level support.
13	DOI 10.1016/j.jpha.2024.101157	Hong et al. (2025). The integration of machine learning into traditional Chinese medicine	DOI registration record matched	Record traceability does not establish claim-level support.
14	DOI 10.15212/AMM-2025-0045	Yin et al. (2025). Artificial intelligence in Traditional Chinese Medicine: systematic insights from data mining, large language models, and multimodal fusion	DOI registration record matched	Record traceability does not establish claim-level support.
15	DOI 10.3389/fphar.2025.1687681	Li et al. (2025). Application and research progress on artificial intelligence in the quality of Traditional Chinese Medicine	DOI registration record matched	Record traceability does not establish claim-level support.
16	DOI 10.1007/s10462-026-11513-w	Wu et al. (2026). Artificial intelligence in Traditional Chinese Medicine herbs: a survey	DOI registration record matched	Record traceability does not establish claim-level support.

Ref.	Identifier / record	Bibliographic record	Check scope	Version / evidence boundary
17	DOI 10.1039/d4sc04107k	Song et al. (2024). AI empowering traditional Chinese medicine?	DOI registration record matched	Record traceability does not establish claim-level support.
18	DOI 10.3390/ph18071074	Yang et al. (2025). Network Pharmacology-Driven Sustainability: AI and Multi-Omics Synergy for Drug Discovery in Traditional Chinese Medicine	DOI registration record matched	Record traceability does not establish claim-level support.
19	DOI 10.3389/fphar.2024.1303693	Wang et al. (2024). A critical assessment of Traditional Chinese Medicine databases as a source for drug discovery	DOI registration record matched	Record traceability does not establish claim-level support.
20	DOI 10.1080/17460441.2021.1909567	Jiménez-Luna et al. (2021). Artificial intelligence in drug discovery: recent advances and future perspectives	DOI registration record matched	Record traceability does not establish claim-level support.
21	DOI 10.1021/acs.chemrev.8b00728	Yang et al. (2019). Concepts of Artificial Intelligence for Computer-Assisted Drug Discovery	DOI registration record matched	Record traceability does not establish claim-level support.
22	DOI 10.1007/s11030-021-10217-3	Gupta et al. (2021). Artificial intelligence to deep learning: machine intelligence approach for drug discovery	DOI registration record matched	Record traceability does not establish claim-level support.
23	DOI 10.1155/sci5/1688637	Sharma et al. (2025). Computational Landscape in Drug Discovery: From AI/ML Models to Translational Application	DOI registration record matched	Record traceability does not establish claim-level support.
24	DOI 10.1021/acs.jcim.2c01073	van Tilborg et al. (2022). Exposing the Limitations of Molecular Machine Learning with Activity Cliffs	DOI registration record matched	Record traceability does not establish claim-level support.
25	DOI 10.1021/ci100050t	Rogers et al. (2010). Extended-Connectivity Fingerprints	DOI registration record matched	Record traceability does not establish claim-level support.
26	https://proceedings.mlr.press/v70/gilmer17a.html	Gilmer et al. (2017). Neural Message Passing for Quantum Chemistry. Proceedings of Machine Learning Research 70, 1263–1272.	Official PMLR proceedings record checked	ICML 2017 proceedings record; matched to Ref. 26 in the main manuscript. Record identity does not establish claim-level support.
27	https://openreview.net/forum?id=6K2RM6wVqKu	27. Zhou, G.; Gao, Z.; Ding, Q.; Zheng, H.; Xu, H.; Wei, Z.; Zhang, L.; Ke, G. Uni-Mol: A Universal 3D Molecular Representation Learning Framework. In Proceedings of ICLR 2023. https://openreview.net/forum?id=6K2RM6wVqKu.	Official proceedings, repository, or catalogue record	Cited ICLR 2023 version; benchmark details from author supplementary material.
28	DOI 10.1002/advs.202504867	Yang et al. (2025). Q-GEM: Quantum Chemistry Knowledge Fusion Geometry-Enhanced Molecular Representation for Property Prediction	DOI registration record matched	Record traceability does not establish claim-level support.
29	https://proceedings.neurips.cc/paper/2020/hash/94aef38441efa3380a3bed3faf1f9d5d-Abstract.html	29. Rong, Y.; Bian, Y.; Xu, T.; Xie, W.; Wei, Y.; Huang, W.; Huang, J. Self-Supervised Graph Transformer on Large-Scale Molecular Data. Advances in Neural Information Processing Systems 2020, 33, 12559–12571. https://proceedings.neurips.cc/paper/2020/hash/94aef38441efa3380a3bed3faf1f9d5d-Abstract.html.	Official proceedings, repository, or catalogue record	Record traceability does not establish claim-level support.
30	DOI 10.1038/s42256-022-00580-7	Ross et al. (2022). Large-scale chemical language representations capture molecular structure and properties	DOI registration record matched	Record traceability does not establish claim-level support.
31	https://arxiv.org/abs/2410.20711v2	31. Li, R.; Liu, W.; Zhou, X.; Li, M.; Zhang, Q.; Chen, H.; Lin, X. Contextual Representation Anchor Network to Alleviate Selection Bias in Few-Shot Drug Discovery. arXiv 2024, arXiv:2410.20711v2 (preprint). https://arxiv.org/abs/2410.20711v2.	Official proceedings, repository, or catalogue record	Preprint status and cited version are explicit; not treated as peer-reviewed clinical evidence.

Ref.	Identifier / record	Bibliographic record	Check scope	Version / evidence boundary
32	https://arxiv.org/abs/2604.16586	32. Li, Z.; Chen, X.; Wen, H.; Zhang, R.Q.; Li, M.; Zhang, X.; Yin, H.; Yang, Q.; Lam, K.-Y.; Lio, P.; et al. A Systematic Survey and Benchmark of Deep Learning for Molecular Property Prediction in the Foundation Model Era. arXiv 2026, arXiv:2604.16586 (preprint). https://arxiv.org/abs/2604.16586.	Official proceedings, repository, or catalogue record	Preprint status and cited version are explicit; not treated as peer-reviewed clinical evidence.
33	https://openreview.net/forum?id=qYceFeHgm4	33. Li, Y.; Kong, L.; Du, Y.; Yu, Y.; Zhuang, Y.; Mu, W.; Zhang, C. MUBen: Benchmarking the Uncertainty of Molecular Representation Models. Transactions on Machine Learning Research 2024. https://openreview.net/forum?id=qYceFeHgm4.	Official proceedings, repository, or catalogue record	Record traceability does not establish claim-level support.
34	DOI 10.3390/foods14040608	Jiang et al. (2025). Advances in the Identification Methods of Food-Medicine Homologous Herbal Materials	DOI registration record matched	Record traceability does not establish claim-level support.
35	DOI 10.14445/22312803/IJCTT-V73I4P123	P et al. (2025). Identification of Medicinal Plants Using Machine Learning Algorithms	DOI registration record matched	Record traceability does not establish claim-level support.
36	DOI 10.1371/journal.pone.0156327	Sun et al. (2016). Chinese Herbal Medicine Image Recognition and Retrieval by Convolutional Neural Network	DOI registration record matched	Record traceability does not establish claim-level support.
37	DOI 10.1371/journal.pone.0344262	Zhu et al. (2026). Toxic chinese herbal medicine recognition in real-world images via multi-scale and attention-enhanced EfficientNetV2	DOI registration record matched	Record traceability does not establish claim-level support.
38	DOI 10.1145/3448218.3448221	Wang et al. (2021). Identifying Chinese Herbal Medicine by Image with Three Deep CNNs	DOI registration record matched	Record traceability does not establish claim-level support.
39	https://arxiv.org/abs/2505.02147v1	39. Thapa, P.; Sharma, M.; Nyachhyon, J.; Pandeya, Y.R. Local Herb Identification Using Transfer Learning: A CNN-Powered Mobile Application for Nepalese Flora. arXiv 2025, arXiv:2505.02147v1 (preprint). https://arxiv.org/abs/2505.02147v1.	Official proceedings, repository, or catalogue record	Preprint status and cited version are explicit; not treated as peer-reviewed clinical evidence.
40	DOI 10.3389/fpls.2025.1672394	Zou et al. (2025). AI-powered recognition of Chinese medicinal herbs with semantic structure modeling and gradient-guided enhancement	DOI registration record matched	Record traceability does not establish claim-level support.
41	DOI 10.1002/pca.3437	Sha et al. (2025). HerbMet: Enhancing metabolomics data analysis for accurate identification of Chinese herbal medicines using deep learning	DOI registration record matched	Record traceability does not establish claim-level support.
42	DOI 10.3389/fchem.2025.1620154	You et al. (2025). Progress in the application of hyperspectral imaging technology in quality detection and in the modernization of Chinese herbal medicines	DOI registration record matched	Record traceability does not establish claim-level support.
43	DOI 10.3390/plants14213365	Yang et al. (2025). Research on Geographical Origin Traceability of Salvia miltiorrhiza by Combining Two-Trace Two-Dimensional (2T2D) Correlation Spectroscopy and Improved DeiT Model	DOI registration record matched	Record traceability does not establish claim-level support.
44	DOI 10.3390/s24165438	Ren et al. (2024). Comparative Analysis of Machine Learning and Deep Learning Algorithms for Assessing Agricultural Product Quality Using NIRS	DOI registration record matched	Record traceability does not establish claim-level support.
45	DOI 10.1016/j.jfca.2025.108041	Zhang et al. (2025). MT-NIRNet: A multi-task deep learning framework for classifying polygonatum origins and varieties based on near-infrared spectroscopy	DOI registration record matched	Record traceability does not establish claim-level support.

Ref.	Identifier / record	Bibliographic record	Check scope	Version / evidence boundary
46	DOI 10.2196/87237	Peng et al. (2026). Application of a Large Visual Language Model on Tongue Image Description Generation and Physical Constitution Reasoning in Traditional Chinese Medicine (TongueVLM): Model Development and Validation Study	DOI registration record matched	Record traceability does not establish claim-level support.
47	DOI 10.1016/j.microc.2025.112697	Cui et al. (2025). A novel strategy for rapid quantification of multiple quality markers and authenticity identification based on near-infrared spectroscopy and machine learning algorithms, Fructus Gardeniae as a case study	DOI registration record matched	Record traceability does not establish claim-level support.
48	DOI 10.1097/ST9.0000000000000079	Bai et al. (2025). Deep learning-based fusion of color and spectral features from hyperspectral imaging for the origin identification of Salvia miltiorrhiza	DOI registration record matched	Record traceability does not establish claim-level support.
49	DOI 10.3390/molecules28176427	Wang et al. (2023). Identification of Turtle-Shell Growth Year Using Hyperspectral Imaging Combined with an Enhanced Spatial–Spectral Attention 3DCNN and a Transformer	DOI registration record matched	Record traceability does not establish claim-level support.
50	DOI 10.3390/e25060847	Zhou et al. (2023). Recognition of Ellipsoid-like Herbaceous Tibetan Medicinal Materials Using DenseNet with Attention and ILBP-Encoded Gabor Features	DOI registration record matched	Record traceability does not establish claim-level support.
51	DOI 10.3390/molecules31050881	Yang et al. (2026). A Novel Strategy for Rapid Quantification of Multiple Quality Indicators and Grade Discrimination of Atractylodis macrocephalae Rhizoma Based on Electronic Nose, Electronic Tongue and Machine-Learning Algorithms	DOI registration record matched	Record traceability does not establish claim-level support.
52	DOI 10.3390/molecules31071140	Shi et al. (2026). Artificial Intelligence-Enabled Intelligent Sensory Systems for Quality Evaluation of Traditional Chinese Medicine: A Review of Electronic Nose, Electronic Tongue, and Machine Vision Approaches	DOI registration record matched	Record traceability does not establish claim-level support.
53	DOI 10.3390/s24010106	Huang et al. (2024). Application of Data Fusion in Traditional Chinese Medicine: A Review	DOI registration record matched	Record traceability does not establish claim-level support.
54	DOI 10.1016/j.compbiomed.2024.108734	Gangwal et al. (2024). Current strategies to address data scarcity in artificial intelligence-based drug discovery: A comprehensive review	DOI registration record matched	Record traceability does not establish claim-level support.
55	DOI 10.3390/diagnostics15243241	Alshammeri et al. (2025). Explainable Cluster-Based Predictive Framework for Early Diagnosis of Autism Spectrum Disorder Using Behavioral Biomarkers	DOI registration record matched	Record traceability does not establish claim-level support.
56	DOI 10.21203/rs.3.rs-7793831/v1	Miguel-Medina et al. (2025). Comparative Evaluation of SHAP and LIME for Clinical Interpretability in Postoperative Cardiac Surgery Mortality Prediction Models	DOI registration record matched	Preprint status and cited version are explicit; not treated as peer-reviewed clinical evidence.
57	https://arxiv.org/abs/2402.03970v3	57. Zabërgja, G.; Kadra, A.; Frey, C.M.M.; Grabocka, J. Tabular Data: Is Deep Learning All You Need? arXiv 2025, arXiv:2402.03970v3 (preprint). https://arxiv.org/abs/2402.03970v3.	Official proceedings, repository, or catalogue record	Preprint status and cited version are explicit; not treated as peer-reviewed clinical evidence.
58	DOI 10.1039/c9sc04336e	Zeng et al. (2020). Target identification among known drugs by deep learning from heterogeneous networks	DOI registration record matched	Record traceability does not establish claim-level support.
59	DOI 10.1038/s44385-024-00003-9	Liao et al. (2025). Application of Artificial Intelligence In Drug-target Interactions Prediction: A Review	DOI registration record matched	Record traceability does not establish claim-level support.
60	DOI 10.3390/ijms24076573	Koutroumpa et al. (2023). A Systematic Review of Deep Learning Methodologies Used in the Drug Discovery Process with Emphasis on In Vivo Validation	DOI registration record matched	Record traceability does not establish claim-level support.

Ref.	Identifier / record	Bibliographic record	Check scope	Version / evidence boundary
61	DOI 10.1186/s13321-024-00816-1	Jimenes-Vargas et al. (2024). Prediction of compound-target interaction using several artificial intelligence algorithms and comparison with a consensus-based strategy	DOI registration record matched	Record traceability does not establish claim-level support.
62	DOI 10.3390/ijms22031392	Verma et al. (2021). SSnet: A Deep Learning Approach for Protein-Ligand Interaction Prediction	DOI registration record matched	Record traceability does not establish claim-level support.
63	DOI 10.1002/advs.202100832	Friedrich et al. (2021). Learning from Nature: From a Marine Natural Product to Synthetic Cyclooxygenase-1 Inhibitors by Automated De Novo Design	DOI registration record matched	Record traceability does not establish claim-level support.
64	DOI 10.1016/j.bmc.2017.06.045	Classen et al. (2018). Complex molecules, clever solutions – Enzymatic approaches towards natural product and active agent syntheses	DOI registration record matched	Record traceability does not establish claim-level support.
65	DOI 10.1038/s44160-023-00358-8	Li et al. (2023). Chemoenzymatic approaches for exploring structure–activity relationship studies of bioactive natural products	DOI registration record matched	Record traceability does not establish claim-level support.
66	DOI 10.1038/nchembio.118	Hopkins et al. (2008). Network pharmacology: the next paradigm in drug discovery	DOI registration record matched	Record traceability does not establish claim-level support.
67	DOI 10.1186/s13020-024-01056-z	Zhai et al. (2025). Network pharmacology: a crucial approach in traditional Chinese medicine research	DOI registration record matched	Record traceability does not establish claim-level support.
68	DOI 10.1186/1752-0509-5-s1-s10	Li et al. (2011). Network target for screening synergistic drug combinations with application to traditional Chinese medicine	DOI registration record matched	Record traceability does not establish claim-level support.
69	DOI 10.1016/j.pharmthera.2016.02.004	Zhou et al. (2016). Effect of Liuwei Dihuang decoction, a traditional Chinese medicinal prescription, on the neuroendocrine immunomodulation network	DOI registration record matched	Record traceability does not establish claim-level support.
70	DOI 10.3389/fphar.2025.1575270	Dai et al. (2025). Network pharmacology and experimental validation to elucidate the pharmacological mechanisms of OATF against kidney stones	DOI registration record matched	Record traceability does not establish claim-level support.
71	DOI 10.1016/j.intimp.2025.115436	Zhang et al. (2025). Exploring the alleviating effect and mechanism of the diterpenoid alkaloid Polyschistine D on rheumatoid arthritis based on network pharmacology and non-targeted metabolomics	DOI registration record matched	Record traceability does not establish claim-level support.
72	https://arxiv.org/abs/2305.17866	72. Liu, J.; Zhuo, H.H.; Jin, K.; Yuan, J.; Yang, Z.; Yao, Z. Sequential Condition Evolved Interaction Knowledge Graph for Traditional Chinese Medicine Recommendation. arXiv 2023, arXiv:2305.17866 (preprint). https://arxiv.org/abs/2305.17866.	Official proceedings, repository, or catalogue record	Preprint status and cited version are explicit; not treated as peer-reviewed clinical evidence.
73	https://arxiv.org/abs/2504.20118	73. He, J.; Guo, Y.; Lam, L.K.; Leung, W.; He, L.; Jiang, Y.; Wang, C.C.; Xing, G.; Chen, H. OpenTCM: A GraphRAG-Empowered LLM-Based System for Traditional Chinese Medicine Knowledge Retrieval and Diagnosis. arXiv 2025, arXiv:2504.20118 (preprint). https://arxiv.org/abs/2504.20118.	Official proceedings, repository, or catalogue record	Preprint status and cited version are explicit; not treated as peer-reviewed clinical evidence.
74	DOI 10.1016/j.jpha.2025.101342	Zeng et al. (2025). Quantifying compatibility mechanisms in traditional Chinese medicine with interpretable graph neural networks	DOI registration record matched	Record traceability does not establish claim-level support.
75	DOI 10.1016/j.csbj.2024.04.016	Ren et al. (2024). TCMM: A unified database for traditional Chinese medicine modernization and therapeutic innovations	DOI registration record matched	Record traceability does not establish claim-level support.
76	DOI 10.1093/nar/gky987	Xu et al. (2019). ETCM: an encyclopaedia of traditional Chinese medicine	DOI registration record matched	Record traceability does not establish claim-level support.

Ref.	Identifier / record	Bibliographic record	Check scope	Version / evidence boundary
77	DOI 10.1093/bib/bbae414	Duan et al. (2024). HTINet2: herb–target prediction via knowledge graph embedding and residual-like graph neural network	DOI registration record matched	Record traceability does not establish claim-level support.
78	DOI 10.1101/gr.1239303	Shannon et al. (2003). Cytoscape: A Software Environment for Integrated Models of Biomolecular Interaction Networks	DOI registration record matched	Record traceability does not establish claim-level support.
79	DOI 10.1093/bioinformatics/btp101	Bindea et al. (2009). ClueGO: a Cytoscape plug-in to decipher functionally grouped gene ontology and pathway annotation networks	DOI registration record matched	Record traceability does not establish claim-level support.
80	DOI 10.1093/nar/gkac1000	Szklarczyk et al. (2023). The STRING database in 2023: protein–protein association networks and functional enrichment analyses for any sequenced genome of interest	DOI registration record matched	Record traceability does not establish claim-level support.
81	DOI 10.1038/s41596-018-0103-9	Reimand et al. (2019). Pathway enrichment analysis and visualization of omics data using g:Profiler, GSEA, Cytoscape and EnrichmentMap	DOI registration record matched	Record traceability does not establish claim-level support.
82	DOI 10.1038/s41467-019-09234-6	Zhou et al. (2019). Metascape provides a biologist-oriented resource for the analysis of systems-level datasets	DOI registration record matched	Record traceability does not establish claim-level support.
83	DOI 10.1093/nar/gkad926	Kong et al. (2024). BATMAN-TCM 2.0: an enhanced integrative database for known and predicted interactions between traditional Chinese medicine ingredients and target proteins	DOI registration record matched	Record traceability does not establish claim-level support.
84	DOI 10.1016/j.heliyon.2024.e39245	Yang et al. (2024). Analysis of medication rule of Traditional Chinese Medicine in treating depression based on data mining	DOI registration record matched	Record traceability does not establish claim-level support.
85	DOI 10.3389/fphar.2020.590824	Wang et al. (2021). Network Patterns of Herbal Combinations in Traditional Chinese Clinical Prescriptions	DOI registration record matched	Record traceability does not establish claim-level support.
86	DOI 10.1007/s13755-022-00207-6	Jin et al. (2023). Meta-path guided graph attention network for explainable herb recommendation	DOI registration record matched	Record traceability does not establish claim-level support.
87	DOI 10.1109/ICDE48307.2020.00020	87. Jin, Y.; Zhang, W.; He, X.; Wang, X.; Wang, X. Syndrome-Aware Herb Recommendation with Multi-Graph Convolution Network. In Proceedings of the 2020 IEEE 36th International Conference on Data Engineering (ICDE); April 2020; pp. 145–156, doi:10.1109/ICDE48307.2020.00020.	Official proceedings, repository, or catalogue record	Record traceability does not establish claim-level support.
88	DOI 10.1016/j.ymeth.2022.05.003	Liu et al. (2022). HPE-GCN: Predicting efficacy of tonic formulae via graph convolutional networks integrating traditionally defined herbal properties	DOI registration record matched	Record traceability does not establish claim-level support.
89	DOI 10.1109/BIBM62325.2024.10822451	89. Zhou, X.; Dong, X.; Li, C.; Bai, Y.; Xu, Y.; Cheung, K.C.; See, S.; Song, X.; Zhang, R.; Zhou, X.; et al. TCM-FTP: Fine-Tuning Large Language Models for Herbal Prescription Prediction. In Proceedings of the 2024 IEEE International Conference on Bioinformatics and Biomedicine (BIBM); December 2024; pp. 4092–4097, doi:10.1109/BIBM62325.2024.10822451.	Official proceedings, repository, or catalogue record	Record traceability does not establish claim-level support.
90	DOI 10.1016/j.cmpbup.2024.100158	Yang et al. (2024). TCM-GPT: Efficient pre-training of large language models for domain adaptation in Traditional Chinese Medicine	DOI registration record matched	Record traceability does not establish claim-level support.
91	DOI 10.1016/j.phrs.2024.107530	Dai et al. (2024). TCMChat: A generative large language model for traditional Chinese medicine	DOI registration record matched	Record traceability does not establish claim-level support.
92	DOI 10.1093/jamia/ocae087	Hua et al. (2024). Lingdan: enhancing encoding of traditional Chinese medicine knowledge for clinical reasoning tasks with large language models	DOI registration record matched	Record traceability does not establish claim-level support.

Ref.	Identifier / record	Bibliographic record	Check scope	Version / evidence boundary
93	DOI 10.2196/35239	Zhang et al. (2022). Transformer- and Generative Adversarial Network–Based Inpatient Traditional Chinese Medicine Prescription Recommendation: Development Study	DOI registration record matched	Record traceability does not establish claim-level support.
94	https://arxiv.org/abs/2502.04345v3	94. Yang, Y.; Ma, T.; Li, R.; Zheng, X.; Shan, G. Jingfang: An LLM-Based Multi-Agent System for Precise Medical Consultation and Syndrome Differentiation in Traditional Chinese Medicine. arXiv 2026, arXiv:2502.04345v3 (preprint). https://arxiv.org/abs/2502.04345v3.	Official proceedings, repository, or catalogue record	Preprint status and cited version are explicit; not treated as peer-reviewed clinical evidence.
95	https://aclanthology.org/2022.ccl-1.80/	95. Ren, M.; Huang, H.; Zhou, Y.; Cao, Q.; Bu, Y.; Gao, Y. TCM-SD: A Benchmark for Probing Syndrome Differentiation via Natural Language Processing. In Proceedings of the 21st Chinese National Conference on Computational Linguistics; Chinese Information Processing Society of China: Nanchang, China, 2022; pp. 908–920. https://aclanthology.org/2022.ccl-1.80/.	Official proceedings, repository, or catalogue record	CCL 2022 / ACL Anthology version, pp. 908–920; author names checked against the paper; no Springer DOI attached to this version.
96	DOI 10.1038/s41597-025-04772-9	Wang et al. (2025). TCMEval-SDT: a benchmark dataset for syndrome differentiation thought of traditional Chinese medicine	DOI registration record matched	Record traceability does not establish claim-level support.
97	DOI 10.1016/j.eng.2018.11.008	Zhang et al. (2019). Chinmedomics: A Powerful Approach Integrating Metabolomics with Serum Pharmacochemistry to Evaluate the Efficacy of Traditional Chinese Medicine	DOI registration record matched	Record traceability does not establish claim-level support.
98	DOI 10.3389/fpls.2024.1463666	Ji et al. (2024). Application of metabolomics in quality control of traditional Chinese medicines: a review	DOI registration record matched	Record traceability does not establish claim-level support.
99	DOI 10.1007/s11684-019-0705-9	Zhao et al. (2020). Chinmedomics facilitated quality-marker discovery of Sijunzi decoction to treat spleen qi deficiency syndrome	DOI registration record matched	Record traceability does not establish claim-level support.
100	DOI 10.3389/fmolb.2024.1376345	Zhang et al. (2024). Chinmedomics strategy for elucidating the effects and effective constituents of Danggui Buxue Decoction in treating blood deficiency syndrome	DOI registration record matched	Record traceability does not establish claim-level support.
101	DOI 10.1016/j.pharmthera.2020.107680	Han et al. (2020). Chinmedomics, a new strategy for evaluating the therapeutic efficacy of herbal medicines	DOI registration record matched	Record traceability does not establish claim-level support.
102	DOI 10.1186/s13020-025-01094-1	Ma et al. (2025). Machine learning-assisted analysis of serum metabolomics and network pharmacology reveals the effective compound from herbal formula against alcoholic liver injury	Replacement primary study matched	Case text checked against the primary study; corrects an earlier study mismatch.
103	DOI 10.1155/2013/298183	Chen et al. (2013). Random Forest in Clinical Metabolomics for Phenotypic Discrimination and Biomarker Selection	DOI registration record matched	Record traceability does not establish claim-level support.
104	DOI 10.1016/j.phymed.2025.156853	Zheng et al. (2025). Insights into the functional characteristics of rhubarb (Rheum officinale Baill) treatment on experimental traumatic brain injury through network pharmacology with metagenomics	DOI registration record matched	Record traceability does not establish claim-level support.
105	DOI 10.3390/metabo10090342	Züllig et al. (2020). A Metabolomics Workflow for Analyzing Complex Biological Samples Using a Combined Method of Untargeted and Target-List Based Approaches	DOI registration record matched	Record traceability does not establish claim-level support.
106	DOI 10.1038/s41467-020-20430-7	Cantini et al. (2021). Benchmarking joint multi-omics dimensionality reduction approaches for the study of cancer	DOI registration record matched	Record traceability does not establish claim-level support.

Ref.	Identifier / record	Bibliographic record	Check scope	Version / evidence boundary
107	DOI 10.3390/jcm14010034	Rohun et al. (2025). Metabolomics in Atrial Fibrillation: Unlocking Novel Biomarkers and Pathways for Diagnosis, Prognosis, and Personalized Treatment	DOI registration record matched	Record traceability does not establish claim-level support.
108	DOI 10.1038/s41531-025-01226-2	Lin et al. (2026). Metabolomic profiles of depression in Parkinson's disease patients	DOI registration record matched	Online 11 Dec 2025; version of record 9 Jan 2026; cite volume 12, article 9 (2026).
109	DOI 10.1016/j.jcmgh.2018.04.003	Martin et al. (2018). The Brain-Gut-Microbiome Axis	DOI registration record matched	Record traceability does not establish claim-level support.
110	DOI 10.1016/j.jpba.2024.116313	Zhang et al. (2024). UPLC-Q-TOF-MS/MS combined with machine learning methods for screening quality indicators of Hypericum perforatum L.	DOI registration record matched	Record traceability does not establish claim-level support.
111	DOI 10.1016/j.arabjc.2022.103762	Xie et al. (2022). Untargeted metabolomics analysis to unveil the chemical markers for the differentiation among three Gleditsia sinensis-derived herbal medicines by ultra-high performance liquid chromatography/quadrupole time-of-flight mass spectrometry	DOI registration record matched	Record traceability does not establish claim-level support.
112	DOI 10.1016/j.jpba.2026.117415	Wang et al. (2026). Integrating non-targeted metabolomics and machine learning for comprehensive phytochemical profiling and intelligent discrimination of Acorus tatarinowii and its adulterants	DOI registration record matched	Record traceability does not establish claim-level support.
113	DOI 10.1016/j.chroma.2025.466014	Huang et al. (2025). Fast and accurate discrimination analysis of Angelicae Pubescentis Radix using non-targeted analytical profiles images and two-dimensional convolution neural network	DOI registration record matched	Record traceability does not establish claim-level support.
114	DOI 10.1016/j.phymed.2015.02.002	Guo et al. (2015). Metabolite identification strategy of non-targeted metabolomics and its application for the identification of components in Chinese multicomponent medicine Abelmoschus manihot L.	DOI registration record matched	Record traceability does not establish claim-level support.
115	DOI 10.3390/foods14193442	Lin et al. (2025). Machine Learning and UHPLC–MS/MS-Based Discrimination of the Geographical Origin of Dendrobium officinale from Yunnan, China	DOI registration record matched	Record traceability does not establish claim-level support.
116	DOI 10.1016/j.apsb.2017.04.012	Yang et al. (2017). Approaches to establish Q-markers for the quality standards of traditional Chinese medicines	DOI registration record matched	Record traceability does not establish claim-level support.
117	DOI 10.1097/gscm.0000000000000033	Li et al. (2024). From biological potency absorbed components to artificial intelligence mining: a review of analytical strategies for the discovery and validation of quality marker in traditional Chinese medicine in the past decade	DOI registration record matched	Record traceability does not establish claim-level support.
118	DOI 10.1186/s13020-021-00438-x	Yang et al. (2021). Integrating metabolomic data with machine learning approach for discovery of Q-markers from Jinqi Jiangtang preparation against type 2 diabetes	DOI registration record matched	Record traceability does not establish claim-level support.
119	DOI 10.1016/j.jchromb.2025.124746	Zhou et al. (2025). Integrated untargeted and targeted metabolomics to reveal the mechanisms of herbal medicine HLXLD on Alzheimer's disease	DOI registration record matched	Record traceability does not establish claim-level support.
120	DOI 10.1186/1758-2946-6-13	Ru et al. (2014). TCMSP: a database of systems pharmacology for drug discovery from herbal medicines	DOI registration record matched	Record traceability does not establish claim-level support.
121	DOI 10.1093/nar/gkae1037	Gao et al. (2025). HERB 2.0: an updated database integrating clinical and experimental evidence for traditional Chinese medicine	DOI registration record matched	Record traceability does not establish claim-level support.
122	DOI 10.1093/nar/gkz981	Wang et al. (2020). Therapeutic target database 2020: enriched resource for facilitating research and early development of targeted therapeutics	DOI registration record matched	Record traceability does not establish claim-level support.

Ref.	Identifier / record	Bibliographic record	Check scope	Version / evidence boundary
123	DOI 10.1093/nar/gkx1037	Wishart et al. (2018). DrugBank 5.0: a major update to the DrugBank database for 2018	DOI registration record matched	Record traceability does not establish claim-level support.
124	DOI 10.1093/nar/gky1033	Kim et al. (2019). PubChem 2019 update: improved access to chemical data	DOI registration record matched	Record traceability does not establish claim-level support.
125	DOI 10.1016/j.talanta.2025.127627	Gao et al. (2025). An AI-driven strategy for active compounds discovery and non-destructive quality control in traditional Chinese medicine: A case of Xuefu Zhuyu Oral Liquid	DOI registration record matched	Record traceability does not establish claim-level support.
126	DOI 10.1038/s41597-025-05522-7	Zhang et al. (2025). TCMP-300: A Comprehensive Traditional Chinese Medicinal Plant Dataset for Plant Recognition	DOI registration record matched	Record traceability does not establish claim-level support.
127	DOI 10.3389/fphar.2023.1289901	Fan et al. (2023). Multi-level advances in databases related to systems pharmacology in traditional Chinese medicine: a 60-year review	DOI registration record matched	Record traceability does not establish claim-level support.
128	DOI 10.1038/s41401-024-01353-1	Li et al. (2025). Overview and limitations of database in global traditional medicines: A narrative review	DOI registration record matched	Record traceability does not establish claim-level support.
129	DOI 10.1007/s00894-021-04674-8	Bian et al. (2021). Generative chemistry: drug discovery with deep learning generative models	DOI registration record matched	Record traceability does not establish claim-level support.
130	DOI 10.1016/j.compbiomed.2022.105403	Martinelli et al. (2022). Generative machine learning for de novo drug discovery: A systematic review	DOI registration record matched	Record traceability does not establish claim-level support.
131	DOI 10.3389/fphar.2024.1331062	Gangwal et al. (2024). Generative artificial intelligence in drug discovery: basic framework, recent advances, challenges, and opportunities	DOI registration record matched	Record traceability does not establish claim-level support.
132	DOI 10.3390/molecules29081867	Shen et al. (2024). NIMO: A Natural Product-Inspired Molecular Generative Model Based on Conditional Transformer	DOI registration record matched	Record traceability does not establish claim-level support.
133	DOI 10.1021/acs.jcim.3c01496	Pang et al. (2024). Deep Generative Models in De Novo Drug Molecule Generation	DOI registration record matched	Record traceability does not establish claim-level support.
134	DOI 10.1016/j.drudis.2024.104009	KP Jayatunga et al. (2024). How successful are AI-discovered drugs in clinical trials? A first analysis and emerging lessons	DOI registration record matched	Record traceability does not establish claim-level support.
135	DOI 10.1021/acs.jcim.5b00238	Xu et al. (2015). Deep Learning for Drug-Induced Liver Injury	DOI registration record matched	Record traceability does not establish claim-level support.
136	DOI 10.3389/fenvs.2015.00080	Mayr et al. (2016). DeepTox: Toxicity Prediction using Deep Learning	DOI registration record matched	Record traceability does not establish claim-level support.
137	DOI 10.3389/fphar.2025.1632775	Gheorghita et al. (2025). Machine learning-based drug-drug interaction prediction: a critical review of models, limitations, and data challenges	DOI registration record matched	Record traceability does not establish claim-level support.
138	DOI 10.3390/toxics12060385	Mostafa et al. (2024). Machine Learning to Predict Drug-Induced Liver Injury and Its Validation on Failed Drug Candidates in Development	DOI registration record matched	Record traceability does not establish claim-level support.
139	DOI 10.1038/s41467-025-65690-3	Bergen et al. (2025). A large-scale human toxicogenomics resource for drug-induced liver injury prediction	DOI registration record matched	Record traceability does not establish claim-level support.
140	DOI 10.1186/s13321-025-01063-8	Tran-Nguyen et al. (2025). HERGAI: an artificial intelligence tool for structure-based prediction of hERG inhibitors	DOI registration record matched	Record traceability does not establish claim-level support.

Ref.	Identifier / record	Bibliographic record	Check scope	Version / evidence boundary
141	DOI 10.1038/s41564-018-0337-x	Valles-Colomer et al. (2019). The neuroactive potential of the human gut microbiota in quality of life and depression	DOI registration record matched	Record traceability does not establish claim-level support.
142	DOI 10.1007/s00394-015-1068-x	Silveira et al. (2017). Preventive rather than therapeutic treatment with high fiber diet attenuates clinical and inflammatory markers of acute and chronic DSS-induced colitis in mice	DOI registration record matched	Record traceability does not establish claim-level support.
143	DOI 10.3390/molecules21030298	Gao et al. (2016). Rhubarb Anthraquinones Protect Rats against Mercuric Chloride ($HgCl_2$)-Induced Acute Renal Failure	Replacement primary study matched	Case text checked against the primary study; corrects an earlier study mismatch.
144	DOI 10.1037/0022-3514.51.6.1173	Baron et al. (1986). The moderator–mediator variable distinction in social psychological research: Conceptual, strategic, and statistical considerations.	DOI registration record matched	Record traceability does not establish claim-level support.
145	DOI 10.1017/CBO9780511803161	Pearl et al. (2009). Causality	DOI registration record matched	Record traceability does not establish claim-level support.
146	DOI 10.1177/0141076814562718	Hill et al. (2015). The environment and disease: association or causation?	DOI registration record matched	Record traceability does not establish claim-level support.
147	DOI 10.1038/s41392-026-02631-6	Liu et al. (2026). Multi-omics and artificial intelligence for precision drug discovery and potential clinical applications	DOI registration record matched	Record traceability does not establish claim-level support.

Table S4. Database snapshots, entity definitions, and counting rules

Database	Version / snapshot	Snapshot note date	Verified counts	Counting rule	Primary source	Comparability boundary
TCMSP	Original database paper (2014)	7 Sep 2026	499 herbs; 29,384 ingredient records; 3,311 targets; 837 diseases	Paper-level component counts; ingredient records are not asserted to be unique stereochemically resolved structures.	Ru et al., J Cheminform 2014, DOI 10.1186/1758-2946-6-13	Ingredient records are not necessarily unique stereochemically resolved molecules.
HERB 2.0	HERB 2.0 paper / database snapshot (2025 issue)	7 Sep 2026	6,892 herbs; 44,595 ingredients; 6,743 formulae; 15,515 targets; 30,170 diseases	Counts follow database component categories in the HERB 2.0 paper; relationships and curated evidence are separate record classes.	Nucleic Acids Res., DOI 10.1093/nar/gkae1037	Use the formal HERB 2.0 snapshot; relationship counts are different units.
BATMAN-TCM 2.0	BATMAN-TCM 2.0 paper	7 Sep 2026	54,832 formulae; 8,404 herbs; 39,171 ingredients; 17,068 known and 2,319,272 predicted ingredient-target interactions	Entity counts and known/predicted interaction counts are reported separately.	Nucleic Acids Res., DOI 10.1093/nar/gkad926	Known and predicted ingredient–target interactions must remain separate.
COCONUT 2.0	Archived Zenodo release (2024)	7 Sep 2026	695,133 molecules in the archived release	Release-specific count following the source’s structure-normalization rules.	Zenodo DOI 10.5281/zenodo.13692394	Use this archived release for reproducible comparisons; live website counts are not a fixed denominator.
NPASS 3.0	NPASS 3.0 database paper (2026 issue)	7 Sep 2026	204,023 natural products; 48,940 organisms; 8,764 targets; 1,048,756 experimental activity records	Natural-product entities, organism entities, targets, and activity records are distinct denominators.	Nucleic Acids Res. 2026, DOI 10.1093/nar/gkaf1196	Distinguish natural-product, organism, target, and activity-record units.
ETCM v2.0	ETCM v2.0 paper (2023)	7 Sep 2026	48,442 formulae; 9,872 Chinese patent drugs; 2,079 medicinal materials; 38,298 ingredients; 1,040 targets; 8,045 diseases	Counts follow ETCM's six data sections; formulas and patent drugs are not herbs or compounds.	Zhang et al., DOI 10.1016/j.apsb.2023.03.012	Formulae, patent drugs, medicinal materials, and ingredients are different entities.
SymMap	Original SymMap paper (2019 issue)	7 Sep 2026	1,717 TCM symptoms; 961 modern-medicine symptoms; 499 herbs; 19,595 ingredients; 4,302 targets; 5,235 diseases	Entity totals are component counts; association counts are reported separately.	Wu et al., Nucleic Acids Res., DOI 10.1093/nar/gky1021	Entity totals and association counts have different denominators.
TCM-ID	Original paper / official site (last update Oct 2020)	7 Sep 2026	Original snapshot: 1,588 prescriptions; 1,313 herbs; 5,669 ingredients; 3,725 three-dimensional structures	TCM-ID is distinct from TCM Database@Taiwan. Counts refer to records in the historical TCM-ID release.	Ji et al., DOI 10.1016/j.jep.2005.11.003; https://bidd.group/TCMID/	TCM-ID and TCM Database@Taiwan are distinct resources.

Table S5. GraphAI counts, denominators, and evidence provenance

Quantity	Source-reported value	Definition / calculation	Evidence location	Interpretation boundary
Chemical-module total	123,647 unique molecules	InChIKey-deduplicated chemical module integrating CHP/PO compounds and broader drug–target resources, including DrugBank and TTD.	Primary Methods 2.5; Results 3.1	Verified as a module-wide count.
Diffusion analysis universe	62,830 TCM-derived compounds	The paper calls these TCM-derived compounds used in the target-coverage analysis; it does not call them the complete set of records with usable structures.	Primary Results 3.2 / Fig. 2A	Different scope from the full chemical module; not the complete count of all structurally usable records.
Known-target subset	7,631; article reports 12.0%	7,631 / 62,830 = 12.145%, which would round to 12.1% at one decimal place.	Primary article, Fig. 2A text; arithmetic audit	12.145% is a calculation from the printed counts, not the source-reported 12.0%.
Post-diffusion covered compounds	62,744; article reports 98.7%	62,744 / 62,830 = 99.863%, not 98.7%.	Primary article, Fig. 2E text; arithmetic audit	99.863% is a calculation from the printed counts, not the source-reported 98.7%.
Difference between module and analysis scopes	123,647 − 62,830 = 60,817	The full module includes broader compounds; no record-level crosswalk allocates the entire 60,817-record difference.	Primary article and Zenodo record 13763953	Do not label all difference records non-TCM, unusable, or excluded for one specific reason.
Association expansion	343,373 to 6,526,940	343,373 pre-existing recorded links; 6,526,940 retained recorded/predicted links after model filtering.	Primary article, Fig. 2E/F text	Neither the final set nor every pre-existing link is established as a direct binding measurement.
Computational share of final associations	6,183,567 of 6,526,940 = 94.74%	6,526,940 − 343,373 = 6,183,567; divide by 6,526,940. Filter: predicted $pK_i > 5$, equivalent to predicted $K_i < 10$ μM.	Primary article, Fig. 2C-F text; arithmetic audit	Report as computationally added predictions, not direct measurements or biochemical confirmation.
CHP-compound provenance	226,589 associations; 31 databases overall	The paper reports graph-level integration from 31 databases but does not provide per-edge source multiplicity. The proportion supported by at least two independent sources is not calculable from aggregate data.	Primary article, TCM-MKG construction and Results	Independent underlying evidence must be traced beyond database names; the unavailable fraction is not zero.

Primary source: Zeng J, Jia X. Quantifying compatibility mechanisms in traditional Chinese medicine with interpretable graph neural networks. J Pharm Anal. 2025;15(8):101342. DOI 10.1016/j.jpha.2025.101342; data record: https://zenodo.org/records/13763953.

Table S6. Multi-omics method capabilities and selection conditions

Method	Primary / benchmark source	Source-supported capability	Interpretation boundary	Candidate use	Comparison rule
DIABLO	Architecture/sample pairing: Singh et al., DOI 10.1093/bioinformatics/bty1054; cross-method context: Ref. 106	may support simultaneous supervised feature selection and classification.	linear latent components and tuning burden may limit some high-dimensional settings.	Candidate use when paired multi-omics samples and labeled outcomes are available.	Compare supervision, sample pairing, missingness, interpretation, and computational requirements; no universal rank.
MOFA+	Argelaguet et al., Genome Biol. 2020, DOI 10.1186/s13059-020-02015-1; Ref. 106	unsupervised shared and view-specific latent factors; supports missing observations.	latent factors require biological annotation and do not by themselves establish mechanism.	Candidate use for exploratory structure discovery, including incomplete multi-view data.	Compare supervision, sample pairing, missingness, interpretation, and computational requirements; no universal rank.
SNF	Wang et al., Nat Methods 2014, DOI 10.1038/nmeth.2810; Ref. 106	fuses sample-similarity networks across data types.	fused similarity is not itself a calibrated predictive model and typically needs downstream analysis.	Candidate use for sample clustering/subtype exploration with matched samples.	Compare supervision, sample pairing, missingness, interpretation, and computational requirements; no universal rank.
PIMKL	Manica et al., npj Syst Biol Appl 2019, DOI 10.1038/s41540-019-0086-3	incorporates networks and pathway gene sets through multiple kernels.	performance and interpretation depend on prior-network and kernel specification.	Candidate use for supervised phenotype prediction when relevant pathway priors exist.	Compare supervision, sample pairing, missingness, interpretation, and computational requirements; no universal rank.
netDx	Pai et al., Mol Syst Biol 2019, DOI 10.15252/msb.20188497	integrates patient-similarity networks and can expose selected networks/features.	interpretability depends on how similarity networks are constructed.	Candidate use for patient classification with heterogeneous patient-level data.	Compare supervision, sample pairing, missingness, interpretation, and computational requirements; no universal rank.
Transformer fusion	Generic architecture category; multimodal review DOI 10.1109/TPAMI.2023.3275156	attention can represent cross-modal interactions.	data/compute needs and interpretability are implementation-dependent.	Candidate research use only; no TCM-specific superiority claim.	Compare supervision, sample pairing, missingness, interpretation, and computational requirements; no universal rank.

Table S7. Generative-model and acquisition-route evidence

Model / route	Evidence source	Supported description	Required evaluation	Boundary
NIMO	[132]	Natural-product-related generative model evaluated on structure-guided, activity-oriented, and pocket-conditioned tasks.	Assess chemical validity, novelty, NP-domain relevance, stereochemistry, and activity under the stated source protocol.	Model-level results do not demonstrate route feasibility or clinical utility in TCM.
VAE / GAN / reinforcement-learning categories	[129,130,133] (reviews)	General generative architectures and optimization strategies discussed at review level.	Name the actual implementation, training set, endpoint, and comparator when making a quantitative claim.	No common-benchmark high/low ratings are assigned to whole architecture families.
Total synthesis / semisynthesis	[63,64,65]	Chemical assembly or modification of an available natural precursor.	Precursor access, step count, stereocontrol, yield, purification, scale, and safety.	A predicted accessible structure still needs a route-specific demonstration.
Biotransformation / biosynthesis	[63,64,65]	Enzyme- or whole-cell-supported conversion or production through a biological pathway.	Enzyme function, substrates, pathway completeness, host compatibility, and measured production.	Absence of a known pathway does not establish chemical inaccessibility.
Chemoenzymatic synthesis	[64,65]	Integration of chemical and enzymatic transformations.	Compatibility of stages, intermediates, selectivity, yield, and scale.	Chemical and biosynthetic feasibility are separate axes; neither follows from graph validity.

Chemical validity, synthetic accessibility, biosynthetic feasibility, and measured activity are distinct evaluation axes. No ordinal comparison is made across unmatched datasets, representations, or route definitions.

Table S8. Endpoint-specific toxicity-prediction evidence

Model/framework	Primary/review source	Dataset and validation evidence	Evidence label	Interpretation boundary
RF/MLP DILI models	Ref. 138; DOI 10.3390/toxics12060385	LiverTox-derived human DILI labels; 10-fold cross-validation; RF mean accuracy 0.631; external failed-candidate set 19/22 correctly classified.	Source-supported; endpoint and set-specific.	Cross-validation and the failed-candidate external set answer different questions; neither establishes TCM clinical utility.
ToxPredictor / DILImap	Ref. 139; DOI 10.1038/s41467-025-65690-3	300 compounds in primary human hepatocytes; independent blinded set of 51 compounds; 88% sensitivity (29/33) and 100% specificity (14/14).	Source-supported; class enrichment and threshold are study-specific.	Independent compound-level experiment in human hepatocytes; not a human treatment trial.
HERGAI	Ref. 140; DOI 10.1186/s13321-025-01063-8	Primary source reports the hERG dataset and model evaluation; values remain tied to its split and endpoint definition.	Source-supported only where explicitly reported.	hERG blockade is one safety endpoint; report class imbalance, decision threshold, and external performance separately.
DDI prediction models	Ref. 137; DOI 10.3389/fphar.2025.1632775	Review supports heterogeneity in tasks, data quality, imbalance, and validation limitations.	Review-level methodological context	No single accuracy range is assigned across heterogeneous drug-interaction tasks.

Table S9. Validation, leakage, and provenance reporting checklist

Item	Required report	Minimum diagnostic	Interpretation boundary
Split unit and order	Define the independent unit (molecule, scaffold, formula, patient, batch, or source). Perform the split before learned preprocessing, feature selection, augmentation, or normalization that could use held-out information.	Provide a workflow diagram or code order and list every operation fitted on training data only.	Leakage-free preprocessing is necessary for an unbiased held-out estimate.
Exact duplicates	Canonicalize identifiers/structures and identify exact duplicate records, discordant labels, salts, stereoisomers, and repeated measurements.	Report counts before and after deduplication and the handling rule for conflicts or replicates.	Exact duplicates across splits can inflate apparent performance.
Near-duplicate molecules	Define the representation and similarity calculation used to detect closely related molecules across splits.	Report the maximum cross-split similarity and the number/proportion of test molecules with a training neighbor above the prespecified threshold.	Near duplicates can create memorization-like evaluation even when identifiers differ.
Similarity threshold	Prespecify fingerprint/embedding, metric, threshold, and rationale. Do not imply that one threshold is universal across representations or endpoints.	Show a sensitivity analysis across plausible thresholds where feasible.	The threshold operationalizes overlap; it is not a biological law.
Cross-split overlap	Quantify train-validation, train-test, and validation-test overlap for molecules, scaffolds, formula components, patients, batches, and data sources as applicable.	Provide counts, proportions, and disposition (move, collapse, exclude, group, or retain with sensitivity analysis).	Readers must be able to distinguish leakage from deliberately shared context.
Scaffold-aware evaluation	Define the scaffold algorithm and the treatment of acyclic, multi-scaffold, stereochemical, and salt variants.	Report scaffold counts per split and the fraction of test scaffolds absent from training.	Scaffold splits better probe structural novelty than random allocation, although they remain dataset-specific.
Random cross-validation	State explicitly that folds were randomly allocated and whether repeated measurements or related molecules were grouped.	Compare with a grouped, scaffold, temporal, or external split when novel-structure extrapolation is claimed.	Random cross-validation estimates interpolation under random allocation; it cannot by itself prove generalization to new natural-product scaffolds.
External or temporal validation	Use an independently assembled source, later time window, institution, assay batch, or prospective experiment where possible.	Report distribution shift, exclusions, missingness, recalibration, and all deviations from the development protocol.	External performance tests transportability under the stated shift, not universal generalization.
Applicability domain	Define when a prediction is considered in-domain and quantify predictive uncertainty or abstention.	Report coverage-performance trade-offs and out-of-domain error separately.	A high aggregate score can conceal unreliable predictions for rare natural-product scaffolds.

Item	Required report	Minimum diagnostic	Interpretation boundary
Graph denominators and provenance	Separate predicted additions, recorded links, direct measurements, and analysis subsets. Report the number with independently traceable underlying support where available.	Provide count equations, source identifiers, and unresolved mappings; do not treat two databases importing one experiment as independent.	An unavailable corroboration fraction is not zero; total database number is not per-edge support.
Cross-study evidence assembly	Identify which study supports each node or relation and mark every untested cross-study connection.	Provide references by component and separate association, intervention, and proposed mediation.	The highest stage reached by one component does not validate the whole chain.

Evidence basis: the manuscript's activity-cliff, molecular-property benchmark, uncertainty-benchmark, and split-design sources [24,32,33], applied here as an operational reporting standard. No universal molecular-similarity threshold is imposed; the study must justify and disclose its chosen representation-specific threshold.

Table S10. Source context for selected numerical claims

Location	Quantity / statement	Source	Evaluation context	Interpretation boundary
Section 2.1.2 / Uni-Mol	70.53% / 88.07% below 1 / 2 Å RMSD	[27], author supplementary benchmark table	CASF-2016; 285 complexes; Vina comparator exhaustiveness 8.	No confidence intervals accompany these rates in that table; see S2.
Section 3.1.2 / OpenTCM	3.73 million characters; 68 texts; >48,000 entities; 152,000 triples	[73], arXiv:2504.20118	Corpus and graph counts reported for that preprint.	Textual graph size is not predictive or biological validation.
Section 3.2 / association rules	21 rules; 270 herbs	[84]	Study-specific prescription-mining result.	Co-occurrence does not establish pharmacological interaction.
Section 3.6 / GraphAI	6,080 formulae; 215 COVID-19 formulae; 215 and 17 detected compounds	[74]	Formula dataset, application subset, and two herb detections use different units.	Chemical detection does not validate predicted targets; denominators in S5.
Section 4.3 / HLXLD	32 altered metabolites; targeted category counts	[119], publisher/PubMed abstract	Brain untargeted discovery; targeted measurements in brain and plasma.	Abstract-level design verification; no constituent-resolved or clinical inference.
Section 5.4.3 / rhubarb AKI	Five-day oral pretreatment before $HgCl_2$ injury	[143]	Total extract and separated fractions in rats.	Preventive fraction-level protection, not evidence for every monomer or microbiota mediation.

Table S11. Comparison with recent TCM-AI reviews

The comparison characterizes each review's organizing emphasis. Statements that a topic is not central do not assert that it is absent. Source identifiers allow the scope descriptions to be checked against the cited review; the distinguishing contribution claimed here is the combined operational framework, not exclusivity of any individual theme.

Review / source	Primary scope	Evidence / validation focus	Provenance and feasibility focus	Contribution of the present review
Ref. 11 Artificial intelligence in traditional Chinese medicine: from systems biological mechanism discovery, real-world clinical evidence inference to personalized clinical decision support DOI: 10.1016/s1875-5364(25)60983-6	Systems biology, real-world clinical evidence, and personalized clinical decision support across TCM.	Emphasizes high-quality repositories, knowledge graphs, causal inference, and clinical evidence. A fixed scope/evidence matrix and an operational leakage checklist are not the central framework.	Data quality and domain knowledge are major themes; per-database version, entity mapping, deduplication, and cross-database robustness are not organized as one reporting standard. Strong clinical and decision-support orientation; route-specific synthetic accessibility versus biosynthetic feasibility is not a central organizing distinction.	The present review is narrower: ML-supported bioactive-compound discovery, explicit evidence levels, and auditable reporting boundaries.
Ref. 12 Artificial Intelligence in Traditional Chinese Medicine: Unraveling Herbal Medicine's Mechanisms DOI: 10.34133/research.1224	AI-assisted reconstruction of herbal-medicine mechanisms through network pharmacology, multi-omics, quality control, and clinical translation.	Advocates a computational-prediction-to-experimental-validation workflow and causality-informed models; it does not use the present review's seven-stage evidence labels or split-leakage checklist as its organizing device.	Knowledge graphs, standardization, and multimodal integration are prominent; database provenance fields are not consolidated into the same executable checklist used here. Discusses generative AI and translation; separate chemical, semisynthetic, biotransformation, chemoenzymatic, and biosynthetic routes are not the central comparison axis.	The present review operationalizes evidence boundaries for screening claims rather than primarily proposing a mechanism-reconstruction paradigm.
Ref. 13 The integration of machine learning into traditional Chinese medicine DOI: 10.1016/j.jpha.2024.101157	Representative ML applications in TCM quality control, mechanism research, diagnosis, and treatment.	Summarizes methodological cases, challenges, and future directions; explicit dual classification by TCM scope and highest validation stage is not the central structure.	Data limitations are discussed broadly; the present review adds itemized provenance, preprocessing, deduplication, and cross-database robustness fields. Broad application and precision-medicine perspective; route-specific feasibility distinctions are not the main organizing focus.	The present review concentrates on screening methodology and links every performance or case claim to validation context and evidence ceilings.
Ref. 14 Artificial intelligence in Traditional Chinese Medicine: systematic insights from data mining, large language models, and multimodal fusion DOI: 10.15212/amm-2025-0045	Data mining, large language models, and multimodal fusion across TCM research and practice.	The review is organized by AI modalities and application scenarios. Evidence hierarchy and molecular split-integrity reporting are not its central comparative axes.	Multimodal data integration and standardization are addressed; this review adds database-version and entity-level reproducibility requirements for screening datasets. Emphasizes emerging AI modalities and clinical reasoning; route-specific acquisition feasibility is not the main focus.	The present review complements its technology breadth with a narrower evidence-audit framework for compound discovery.

Review / source	Primary scope	Evidence / validation focus	Provenance and feasibility focus	Contribution of the present review
Ref. 15 Application and research progress on artificial intelligence in the quality of Traditional Chinese Medicine DOI: 10.3389/fphar.2025.1687681	AI and bioinformatics for TCM quality assessment using spectra, chromatograms, images, and text.	Focuses on predictive quality models and practical challenges. The seven-stage pharmacological evidence hierarchy and scaffold-leakage audit are outside its main quality-control framework.	Strong emphasis on high-quality, standardized, representative multi-source data; the present review extends this into explicit provenance and cross-database reporting fields for screening. Quality assurance and clinical safety are emphasized; compound-acquisition route feasibility is not a principal topic.	The present review addresses a different endpoint: bioactive-compound discovery and the evidence needed to interpret target and efficacy claims.
Ref. 16 Artificial intelligence in Traditional Chinese Medicine herbs: a survey DOI: 10.1007/s10462-026-11513-w	AI in TCM herb research, comparing traditional and modern research approaches from expert systems to ML, deep learning, and large models.	Discusses explainability, evidence, data quality, ethics, and traditional-knowledge protection; the present review adds fixed validation stages and an operational split-overlap checklist.	Data quality, knowledge graphs, privacy, and ethics are explicit themes; per-resource counting and edge-provenance rules are not the central comparison framework. Distinctive focus on traditional-versus-modern research logic and future AI platforms; route-specific synthesis and biosynthesis are not central.	The present review is differentiated by screening-specific evidence ceilings, not by a broader survey of all AI-herb research.
Ref. 17 AI empowering traditional Chinese medicine? DOI: 10.1039/d4sc04107k	Broad AI-TCM landscape including databases, ingredient analysis, drug discovery, diagnosis, treatment, network pharmacology, and personalized medicine.	Describes challenges such as standardization and interpretability; it does not organize examples through the present review's fixed scope and validation ladder or leakage-control standard.	TCM databases are surveyed extensively; the present review adds version, access date, mapping, preprocessing, deduplication, and robustness requirements. Covers clinical applications and future opportunities; chemical versus biosynthetic route feasibility is not the main organizing distinction.	The present review trades breadth for explicit methodological and evidentiary auditability in bioactive screening.
Ref. 18 Network Pharmacology-Driven Sustainability: AI and Multi-Omics Synergy for Drug Discovery in Traditional Chinese Medicine DOI: 10.3390/ph18071074	Network-pharmacology, AI, and multi-omics integration for sustainable TCM drug discovery, including a mapping of 7,288 publications and 79 selected cases.	Emphasizes mechanism-based validation and integrated workflows. The present review differs by assigning the highest evidence level to each example and by specifying split leakage and cross-study comparability boundaries.	Highlights heterogeneous data integration and sustainability; the present review supplies a more granular provenance and cross-database reporting checklist. Sustainable discovery and translational integration are central. The present review separately defines synthetic accessibility and biosynthetic feasibility and lists acquisition routes.	Closest topical neighbor. The novelty claimed here is not topic breadth, but evidence grading, validation safeguards, provenance audit, and claim calibration.
Present review	ML methodology for TCM bioactive-compound screening, formula analysis, multi-omics evidence, and drug-development support.	Uses independent scope tiers plus seven evidence stages; adds dataset/split context, leakage and near-duplicate checks, applicability domains, and claim-boundary rules.	Requires versions, access dates, entity mapping, preprocessing, deduplication, counting rules, and cross-database robustness; audits GraphAI denominators and edge provenance. Separates chemical synthesis feasibility from biosynthetic feasibility and identifies total synthesis, semisynthesis, biotransformation, and chemoenzymatic routes.	Contribution is a screening-specific, evidence-calibrated, and operational reporting framework rather than a claim of comprehensive AI-TCM coverage.
